# Five-second coherence of a single spin with single-shot readout in silicon carbide

**DOI:** 10.1126/sciadv.abm5912

**Published:** 2022-02-02

**Authors:** Christopher P. Anderson, Elena O. Glen, Cyrus Zeledon, Alexandre Bourassa, Yu Jin, Yizhi Zhu, Christian Vorwerk, Alexander L. Crook, Hiroshi Abe, Jawad Ul-Hassan, Takeshi Ohshima, Nguyen T. Son, Giulia Galli, David D. Awschalom

**Affiliations:** 1 Pritzker School of Molecular Engineering, University of Chicago, Chicago, IL 60637, USA.; 2 Department of Physics, University of Chicago, Chicago, IL 60637, USA.; 3 Department of Chemistry, University of Chicago, Chicago, IL 60637, USA.; 4 National Institutes for Quantum Science and Technology, 1233 Watanuki, Takasaki, Gunma 370-1292, Japan.; 5 Department of Physics, Chemistry and Biology, Linköping University, SE-581 83 Linköping, Sweden.; 6 Center for Molecular Engineering and Materials Science Division, Argonne National Laboratory, Lemont, IL 60439, USA.

## Abstract

An outstanding hurdle for defect spin qubits in silicon carbide (SiC) is single-shot readout, a deterministic measurement of the quantum state. Here, we demonstrate single-shot readout of single defects in SiC via spin-to-charge conversion, whereby the defect’s spin state is mapped onto a long-lived charge state. With this technique, we achieve over 80% readout fidelity without pre- or postselection, resulting in a high signal-to-noise ratio that enables us to measure long spin coherence times. Combined with pulsed dynamical decoupling sequences in an isotopically purified host material, we report single-spin *T*_2_ > 5 seconds, over two orders of magnitude greater than previously reported in this system. The mapping of these coherent spin states onto single charges unlocks both single-shot readout for scalable quantum nodes and opportunities for electrical readout via integration with semiconductor devices.

## Teaser

Mapping spins onto single charges enables deterministic readout of quantum states and measurement of long coherence times.

## INTRODUCTION

Solid-state defect spins hold promise for use in quantum information processing, sensing, and communication because of their unique combination of long coherence times (*1*–*4*), a spin-photon interface (*5*, *6*), and the availability of nuclear registers for use as robust quantum memories (*7*, *8*). The neutral divacancy (VV^0^) in silicon carbide (SiC) boasts these features with the added advantages of the SiC material platform, including wafer-scale commercial availability; complementary metal-oxide semiconductor (CMOS) compatibility; and the ability to fabricate hybrid photonic (*9*, *10*), electrical (*11*), and mechanical devices (*12*, *13*).

Typically, for optically active defects spins, single-shot readout is performed through spin-dependent fluorescence probed with narrow-line lasers resonant with the defect’s optical cycling transitions (*14*, *15*). However, this method suffers from spin-flip errors due to nonunity branching ratios in the defect’s optical excited state (*5*). As a result, only a finite number of spin-correlated photons are scattered before destroying the state. Combined with poor collection efficiencies, the number of measured photons per shot is usually low (*N* << 1) unless photonic devices are used to enhance emission and collection. Hence, a key hurdle for the divacancy system to date has been the ability to perform single-shot readout of the defect’s spin state (*16*). This single-shot readout unlocks the ability to perform the entanglement distribution (*17*) and quantum error correction (*18*) needed to make quantum networks a reality and provides an increased signal-to-noise ratio for quantum sensing.

Another avenue toward single-shot readout is spin-to-charge conversion (SCC), which maps the defect spin state onto a robust, long-lived charge state. For isolated single defect spins, SCC is an all-optical technique that has been used to achieve high-fidelity single-shot readout but has thus far been limited to demonstrations using the NV^−^ (negatively charged nitrogen vacancy center) in diamond (*19*, *20*). In this work, we demonstrate the first ever implementation of SCC for VV^0^ in SiC by performing spin-selective ionization followed by all-optical single-shot readout of the charge state. Using this technique, we can determine an initially prepared spin state with over 80% fidelity. We note that in this work, we do not pre- or postselect the charge state or resonance condition, a common strategy used to artificially boost fidelity at the cost of success rate (*14*, *19*, *20*). Critically, we also achieve this readout in the absence of photonic enhancement, demonstrating the value of SCC to other systems where nanofabrication remains a challenge or where detector technologies may be limited. In addition, we deepen our understanding of the SCC process for the VV^0^ defect with ab initio density functional theory (DFT) calculations that clarify the charge transition process, which, combined with theoretical modeling, assist with further optimization of this type of readout.

The high-fidelity and single-shot readout provided by the SCC technique enable us to perform experiments that would otherwise be infeasible because of low signal-to-noise ratio, such as when the time per experimental “shot” becomes prohibitively long. With this advantage, we use the SCC technique to probe the unexplored limits of the spin lifetime and coherence time for VV^0^. These times fundamentally determine the divacancy’s performance in future quantum architectures by limiting metrics such as quantum memory times and sensitivity for ac sensing schemes (*21*–*23*). Here, we first establish an experimentally limited lower bound on the spin *T*_1_ time of at least 103 s for VV^0^, over two orders of magnitude longer than previously reported (*7*). In this isotopically purified sample, we combine the reduction of the defect’s noisy nuclear environment with the use of dynamical decoupling sequences to preserve coherence (*24*, *25*). As a result, we measure a *T*_2_ time of 5.3 ± 1.3 s, over three orders of magnitude greater than the natural Hahn echo *T*_2_ time (*3*). These metrics establish VV^0^ as a premier system with coherence times that exceed previous reports for electron spin qubits in both natural and highly isotopically purified silicon (*26*, *27*), diamond (*28*, *29*), and SiC (*2*, *30*).

The results presented in this work develop SiC-based systems as a promising platform for quantum technologies, where both deterministic readout of the spin state and long coherence times are necessary for heralded entanglement generation, high gate fidelities, and the development of network components such as quantum repeaters. This work also opens avenues that use the CMOS compatibility of SiC for the integration of electron spin–based systems in classical electrical devices that are sensitive to single charges.

## RESULTS

### Optical and charge transitions of the divacancy in SiC

**Fig. 1.**
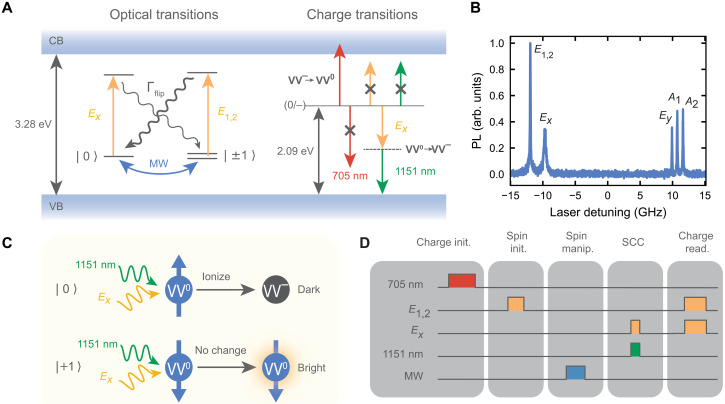
Control and readout of spin and charge states of the divacancy. (**A**) Optical and charge transitions of the divacancy. Excitation of the *E_x_* and *E*_1,2_ spin-selective optical transitions is performed using ~1131-nm light (yellow). Spin-flips (Γ_flip_) prevent indefinite readout of the spin state with laser pumping. Microwave (MW) manipulation is used to induce ground-state spin-sublevel transitions. SCC is performed by excitation of the *E_x_* transition followed by an ejection of a hole by the 1151-nm ionization laser (green). The dashed line represents the VV^0^ excited state. The 705-nm light (red) resets the divacancy from VV^−^ to VV^0^. The individual lasers used for SCC and charge resetting do not have enough energy to induce other charge transitions via a one-photon process (denoted by an “X”). (**B**) PL excitation spectrum of a single divacancy reveals its six spin-selective optical transitions at *T* = 5 K and *B* = 18 G with continuous microwave driving of the *m_s_* = 0↔*m_s_* = +1 transition. Detuning is relative to a center laser frequency of 265.1408 THz, and the transverse strain splitting is 9.78 GHz. The *E_x_* and *E_y_* optical transitions are *m_s_* = 0 character, while the *E*_1,2_, *A*_1_, and *A*_2_ transitions are *m_s_* = ±1 character (*5*). (**C**) Mapping of the spin state onto the charge state. Pumping of the *E_x_* transition allows for ionization of *m_s_* = 0 with the 1151-nm laser. The SCC step is followed by charge readout via pumping of both the *E_x_* and *E*_1,2_ transitions. Detection of PL signifies that the divacancy is in its bright, neutral (dark, ionized) state and therefore was prepared to *m_s_* = +1 (*m_s_* = 0). (**D**) Typical experimental pulse sequence. After the charge and spin initialization and microwave manipulation of the spin state, single-shot readout of the spin state is performed with SCC followed by readout of the charge state.

The neutral divacancy in 4H-SiC is a deep-level defect consisting of a carbon and silicon vacancy pair. The dangling bonds from atoms neighboring these vacancies form a spin-triplet ground state (*31*) with spin sublevels that can be polarized and read out with laser light and manipulated with microwaves (Fig. 1A). In this work, we use laser light resonant with the *E_x_* and *E*_1,2_ spin-selective optical transitions (“resonant excitation”), corresponding to the *m_s_* = 0 and *m_s_* = ±1 spin sublevels (*5*), respectively (Fig. 1B). The divacancy spin state can be efficiently initialized to *m_s_* = 0 via excitation of the *E*_1,2_ transition, which depletes the *m_s_* = ±1 population through optical pumping and nonradiatively polarizes into *m_s_* = 0 via a spin-singlet intersystem crossing (*32*, *33*). In past work, spin-photon readout is performed by collecting photoluminescence (PL) scattered when pumping on one of the more cycling resonant optical lines, such as the *E_x_* transition. However, when pumping on a single optical transition, spin-flips from the excited state (*32*) cause a depopulation that prevents indefinite optical readout. The finite number of photons emitted before destroying the state ultimately limits the fidelity of the spin-photon readout technique.

As an alternative to readout via the spin-photon interface, the divacancy hosts robust charge states (*34*) that can be manipulated and read out using laser light (*11*, *35*, *36*). In this work, we use the fact that non-neutral states of the divacancy do not appreciably photoluminesce under resonant excitation that is tuned to the neutral state’s zero-phonon line (ZPL). When the optical lines corresponding to both *m_s_* = 0 and *m_s_* = ±1 of VV^0^ are simultaneously pumped (“charge readout”), the emitted PL does not reflect the spin state but rather whether the defect is in the neutral state or not, provided that the lasers are on resonance. Thus, a reduction in PL distinguishes “dark” ionized states from the “bright” neutral state (Fig. 1C), as the optical lines are stable in this sample. For the VV^0^ in SiC, this dark state has been established as the negatively charged divacancy (VV^−^) (*11*, *35*–*37*). Crucially, probing the divacancy charge state with this light is not energetic enough to convert VV^−^ to VV^0^ and vice versa via a direct one-photon process (Fig. 1A). The result is that nondestructive measurement of the charge state of the defect is possible with high fidelity. In this work, we also rely on deterministic preparation of the defect into the neutral charge state (VV^0^). Previous reports have shown that laser light above ~1.3 eV resets the charge state from VV^−^ to VV^0^ and that light around 705 nm (1.76 eV) is extremely efficient in charge initializing the divacancy to its neutral state (*11*). However, the fidelity of this process has remained unexplored to date.

Mapping of the spin state onto the charge state (SCC) via a spin-selective two-photon ionization process provides us with an avenue toward performing high-fidelity, deterministic measurement of the spin state via readout of the charge state. Here, we access the defect’s excited state in a one-photon, spin-selective manner using a narrow-line laser tuned to one of the resolved optical transitions (Fig. 1, A and B). A second “ionization laser” (1151 nm) takes the defect from its excited state to the ionized state (VV^−^) via a one-photon process by ejecting a hole (*38*). We select the ionization laser wavelength based on the results of DFT calculations that we perform for the VV^0^ charge transition energies, which, for the (0/−) transition, is calculated to be 2.09 eV, in good agreement with previous work (*31*, *38*). The ionization laser (1151 nm) is red-detuned from the defect’s ZPL (1131 nm) so as not to excite any optical transitions while still providing enough energy to ionize the defect from the excited state, as the combined energy of these photons (2.17 eV) enables the 2.09-eV (0/−) charge transition (*34*, *38*) to occur (Fig. 1A). Specifically, we go beyond this estimate based on the charge transition levels and directly compute using DFT the energy required to go from the VV^0^ optical excited state to the VV^−^ state with a hole at the valence band maximum. Our DFT calculations show that photoionization from the excited state requires ~1.03 eV, an energy that is slightly less than the calculated ZPL of 1.196 eV, in good agreement with experimental results (Supplementary Materials). This means that we can use a narrow, low-power resonant laser tuned to a single optical transition to provide spin selectivity alongside a high-power, red-detuned ionization tone to induce, in total, a two-photon spin-dependent ionization of the defect.

Once we have spin-selectively mapped the spin onto the defect’s charge state with the SCC step, we can then perform single-shot readout of the charge state by addressing the defect with both *E_x_* and *E*_1,2_ resonant light and collecting PL (Fig. 1C). Thus, we are equipped with a full suite of techniques to initialize the charge and spin state on demand, manipulate the spin with microwave pulses, convert the spin state to a charge state, and perform charge readout with these lasers and controls (Fig. 1D).

### Charge control and readout

**Fig. 2.**
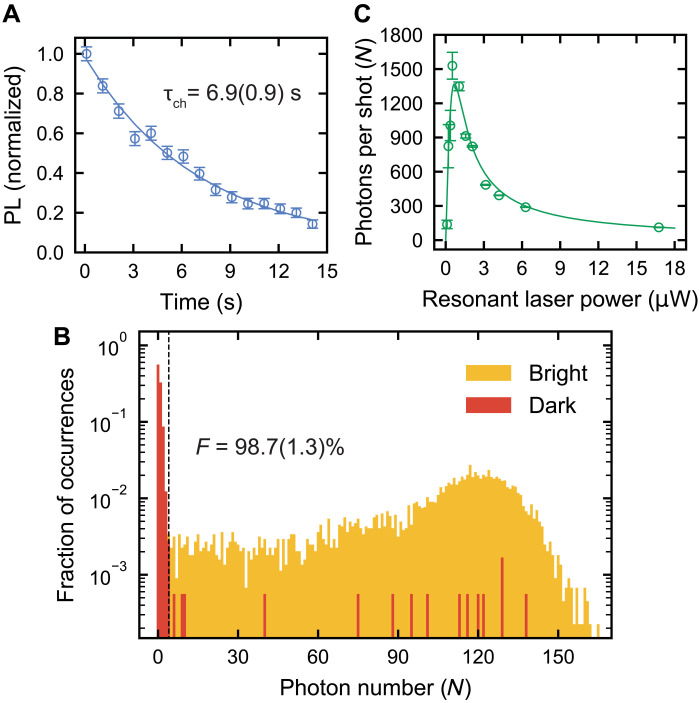
Single-shot readout of the divacancy charge state. (**A**) Charge readout PL signal dependence on delay time between the charge initialization and readout follows an exponential decay $e^{\left(-\frac{t}{\tau_{\text{ch}}}\right)}$, where τ_ch_ is the charge lifetime. We find that τ_ch_ is 6.9(0.9) s. (**B**) Log-scale histogram of photon number distributions collected during a charge readout for preparation into the neutral bright state and ionized dark state. We use a 20-ms readout window with 4.05-μW combined resonant laser power, selected to maximize the readout fidelity. For a cutoff of *N* = 4 photons, the single-shot readout fidelity of the charge state is 98.7(1.3)%. The false-positive rate *p*_0*|*1_ = 1.17%, and false-negative rate *p*_1*|*0_ = 1.26%. (**C**) Extracted photons per shot from observed PL rate and charge state decay for various combined resonant laser powers. The maximal extracted photons per shot is *N* = 1529(117). The line is a fit from a model (Supplementary Materials). All data are taken at *B* = 18 G and *T* = 5 K. All reported errors represent 1 SE from the fit, and all error bars represent 1 SD of the raw data.

We first demonstrate the robustness of the divacancy charge state and our ability to perform single-shot, high-fidelity optical readout of this state. We characterize the longevity of the VV^0^ charge state with a sequence consisting of a 705-nm charge initialization pulse, a variable delay, and an optical charge readout from which we extract a charge lifetime τ_ch_ = 6.9 ± 0.9 s (Fig. 2A and Supplementary Materials). The finite duration of the charge lifetime when the defect is not under illumination is likely due to diffusion of charges from nearby shallow nitrogen donors, as the material is slightly n-type (Materials and Methods). The charge lifetime is a critical time scale that dictates the longest permissible time during an experiment where the neutral defect, and therefore the spin, remains stable. Thus, the charge lifetime ultimately limits the qubit’s lifetime and is a stringent cutoff for sensing and memory applications. Fortunately, previous work has shown much longer charge lifetimes (*35*), where future tuning of the Fermi level or balance of deep traps in the material may extend our measured time scale by many orders of magnitude. This charge instability is also linked to our ability to optically ionize the defect on demand, representing a tradeoff to be optimized in future materials design. Our measured charge lifetime, however, is still many orders of magnitude longer than the saturated spin-flip lifetime, which we measure to be 3.3 ± 0.1 μs (Supplementary Materials), resulting in a longer possible readout window and more scattered photons before destroying the state.

We next demonstrate our ability to perform single-shot readout of the charge state. First, we either charge-initialize to the bright state using a long, 705-nm laser pulse or spin-agnostically initialize to the dark state using an “ionization pulse” where both *E_x_* and *E*_1,2_ resonant lasers and the 1151-nm ionization laser are simultaneously turned on. This preparation into either the bright or dark charge state is followed by optical charge readout. Figure 2B displays the number distribution of photons collected during the charge readout step for both the prepared bright and dark initial states. We calculate that for preparation into the bright (dark) state, the mean photon number is *N* = 100 ± 1 (*N* = 1.3 ± 1.1) (Supplementary Materials). We determine that for a cutoff of *N* = 4 photons, the fidelity, *F*_charge_ (*39*) is maximized at 98.7 ± 1.3% (Supplementary Materials), representing the total fidelity of our ability to prepare and read out the defect’s charge state in a single shot. Despite the absence of solid-immersion lenses and other photonically enhancing structures, we achieve a high number of photons per shot. The nonunity fidelity likely arises from imperfect charge state preparation due to optical excitation of nearby traps, as we discuss in the following sections. The high single-shot photon number and the near-unity fidelity of the charge readout technique exemplify its advantage over traditional spin-photon readout.

Figure 2C shows the projected number of photons per shot during these charge readout windows using various resonant laser powers. With increased laser power, more photons are scattered per second, but additional two-photon ionization occurs, reducing the time during which the charge state can be read. On the other hand, if the laser power is too low, then very few photons per second are scattered but readout time is still limited by the charge lifetime and the readout window cannot be arbitrarily extended. As a result, there is an optimal choice of laser power to maximize the readout, which, in our case, results in over 1500 photons per shot at ~1 μW. This behavior is understood with a simple predictive model (Supplementary Materials). The high number of scattered photons means that single-shot readout of the charge state is possible even with extremely low collection efficiency or in systems with much lower quantum yield.

**Fig. 3.**
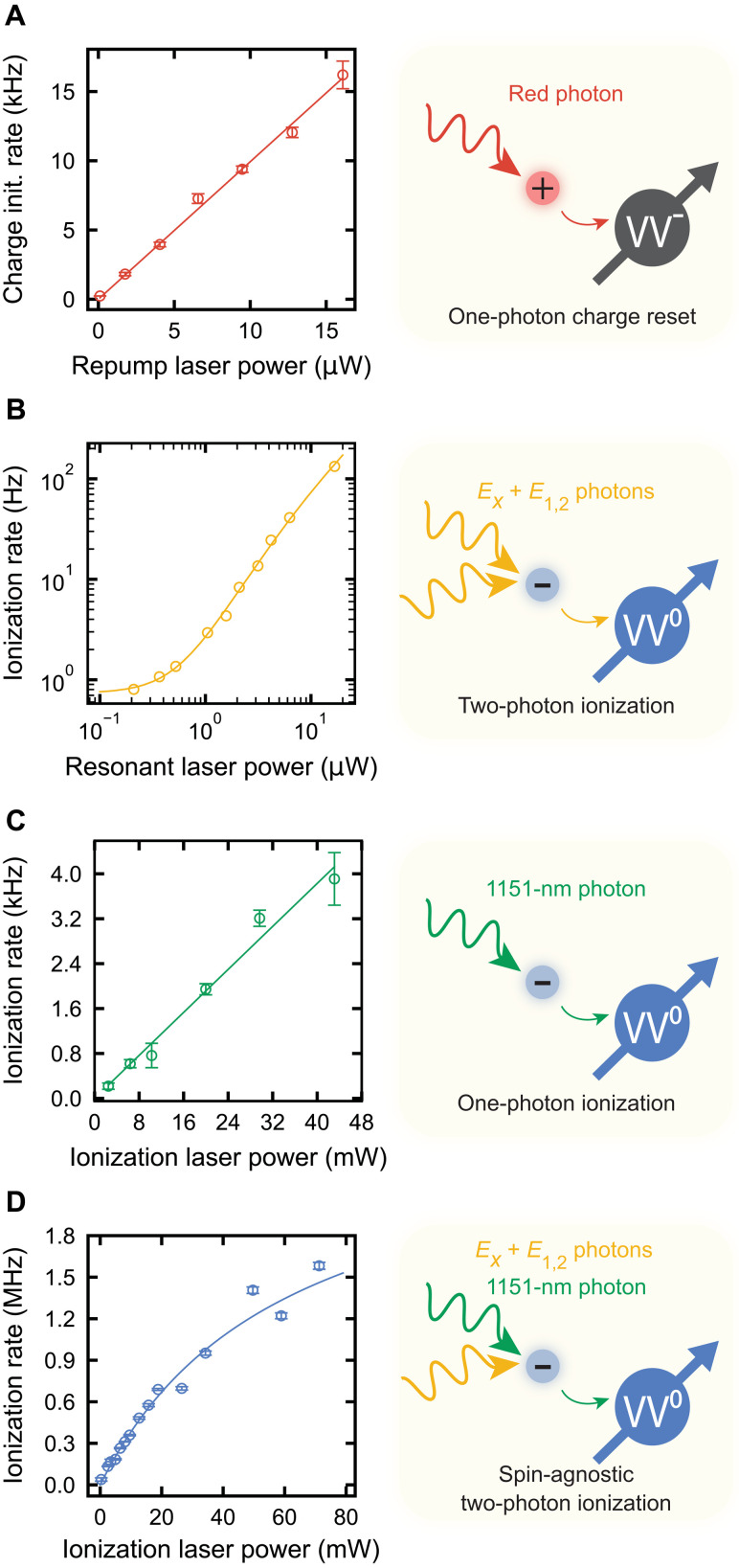
Optical charge reset and ionization processes. (**A**) Power dependence of the charge reset rate using the 705-nm laser. The reset rate is linear (red line fit) with power as 993 ± 17 Hz/μW. (**B**) Ionization rate dependence on combined resonant laser power (*E_x_* and *E*_1,2_ lines). The solid line is a fit using a saturating two-photon ionization model (Supplementary Materials). (**C**) Ionization rate dependence on 1151-nm laser alone. The ionization rate is linear with power (solid line fit) as 95.7 ± 3.7 kHz/W. (**D**) Spin-agnostic ionization rate dependence on the 1151-nm laser ionization power. The resonant laser excitation is beyond saturation at 15 μW. The solid blue line is a fit from our model including the effect of stimulated emission (Supplementary Materials), where the low-power ionization rate is 37.4 ± 0.7 MHz/W. All data are taken at *B* = 18 G and *T* = 5 K. All reported errors represent 1 SE from the fit, and all error bars represent 1 SD of the raw data.

Having established our ability to perform high-fidelity single-shot readout of the charge state, we next characterize the various charge transition processes induced by the lasers used in our experiments. We first characterize the rate at which we initialize the divacancy to the neutral state with the 705-nm charge repump laser. After preparing the defect in the dark charge state, a time-varying charge initialization pulse is applied to reset the defect to the neutral, bright state. Using a charge readout, we measure the recovery rate of the bright state as a function of the 705-nm laser power (Fig. 3A). The charge repump rate is 993 ± 17 MHz/W, consistent with the one-photon repumping rate described in (*11*).

We next characterize the ionization rate solely from the resonant lasers and ionization laser, respectively, by initializing into the bright state and measuring the charge state after a variable length laser pulse. The exponentially fit ionization rates for various powers of the resonant lasers are displayed in Fig. 3B. The ionization rate is initially quadratic and then increases linearly as 10.6 ± 0.9 MHz/W, signaling saturation of the optical transition (Supplementary Materials). At the relevant resonant laser powers in our experiment, we observe ionization rates around 100 Hz. On the other hand, ionization from solely the 1151-nm laser increases linearly with power as 95.7 ± 3.7 kHz/W (Fig. 3C). Ideally, the 1151-nm laser by itself should cause no ionization, where, here, a small residual rate likely arises from excitation of nearby traps, freeing carriers that alter the defect’s charge state.

Last, we characterize our ability to ionize the defect once it is in its optical excited state, which is the prerequisite for spin-dependent ionization. After charge-initializing to the bright state, we spin-agnostically ionize the defect using a variable length pulse where both the *E_x_* and *E*_1,2_ resonant lasers and the 1151-nm ionization laser are on at the same time. For these experiments, the resonant power is kept such that the defect optical transition is saturated. The decay rate of the signal from charge readout is displayed for various ionization powers in Fig. 3D, where the saturating behavior can be understood through the effect of stimulated emission, as discussed later (Supplementary Materials).

The spin-agnostic ionization rates (order MHz) are nearly three orders of magnitude greater than the unwanted ionization rate from only the 1151-nm laser (order kHz) or only the resonant lasers (order 100 Hz) for the relevant powers used in our experiments. This confirms that for experiments where both resonant excitation and the 1151-nm ionization laser are used, the dominant source of ionization is a two-photon process where one photon induces a resonant, ground–to–excited state transition of the defect and a second photon from the ionization laser subsequently converts the defect to its negative charge state.

### Spin-to-charge control

Given that we can perform high-fidelity, single-shot readout of the divacancy charge state and that we can ionize the defect with a combination of resonant and red-detuned laser light, we can selectively map the divacancy spin state onto its charge state to achieve single-shot readout of the spin state. Specifically, after charge initialization to VV^0^ and spin initialization to *m_s_* = 0, we spin-selectively photoionize the *m_s_* = 0 state to VV^−^ by simultaneous excitation of the *E_x_* optical transition (*m_s_* = 0 character) while applying the 1151-nm ionization laser. This is the SCC pulse that results in the process shown in Fig. 1C. The defect can be protected from this spin-selective ionization by rotating into *m_s_* = +1 via the application of a microwave π pulse so that the laser no longer optically excites it (fig. S7). Thus, a spin initialized to *m_s_* = +1 does not undergo ionization and remains in VV^0^, forming the basis of spin contrast for the SCC process. SCC is performed using the *E_x_* transition due to its high cyclicity, which increases the number of times the excited state can be populated before a spin-flip occurs. Spin-flips cause destruction of the spin state (*34*) and prevent ionization from occurring, therefore reducing the fidelity of the conversion process (Supplementary Materials). Thus, a key aspect of the SCC process is ensuring that the rate of spin-selective ionization exceeds the rate of spin-flip errors.

**Fig. 4.**
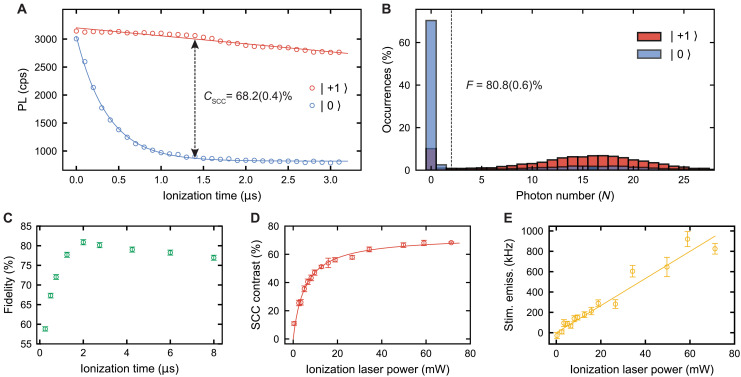
Single-shot readout of the spin state with SCC. (**A**) Charge readout signal following SCC step for preparation into *m_s_* = 0 and *m_s_* = +1. The maximum fitted contrast is 68.2(0.4)% at an ionization laser power of 71 mW and a SCC pulse duration of *t*_ion_ = 1.39 μs using 14.95 μW of resonant power. cps, counts per second. (**B**) Charge readout photon number distribution after SCC step for preparation into *m_s_* = 0 and *m_s_* = +1. The end-to-end process fidelity is 80.8(0.6)% for a cutoff of *N* = 2 photons. (**C**) Dependence of SCC fidelity on SCC pulse duration. (**D**) Dependence of SCC contrast with 1151-nm ionization laser power. The contrast follows a saturation behavior (red line fit; Supplementary Materials). (**E**) Calculated stimulated emission rate dependence on 1151-nm ionization laser power. The stimulated emission rate increases linearly as 13.3 MHz/W (yellow line fit). All data are taken at *B* = 18 G and *T* = 5 K. All reported errors represent 1 SE from the fit, and all error bars represent 1 SD of the raw data.

After SCC, we perform single-shot readout of the charge state. The charge readout signal for states prepared to *m_s_* = 0 and *m_s_* = +1 as the SCC pulse duration is swept is shown in Fig. 4A. After the SCC contrast reaches a maximum, the contrast decreases with increased spin-selective ionization pulse durations due to a reduction in PL from states prepared into *m_s_* = +1. This decreasing PL is caused by non–spin-selective ionization but has a rate of decay that exceeds the ionization rate from only the 1151-nm laser (Fig. 3C). Therefore, we attribute this non–spin-selective ionization to other mechanisms such as weak excitation of the *m_s_* = ±1 optical transitions by the *E_x_* laser or heating effects induced by the high-power ionization laser that cause additional orbital or spin mixing.

We next characterize the end-to-end fidelity of the combined initialization, SCC, and readout process by examining the resulting single-shot photon number distribution. Figure 4B shows histograms of the photon statistics for states prepared into *m_s_* = 0 and *m_s_* = +1. We extract a maximum end-to-end SCC fidelity of *F*_SCC_ = 80.8 ± 0.6% (Supplementary Materials). When corrected for the charge initialization and charge readout fidelity, we obtain an SCC fidelity of *F*_SCC_′ *= F*_SCC_/*F*_charge_ *=* 81.6%, revealing that the main source of infidelity is the SCC conversion process and not the charge readout. We eliminate infidelity arising from errors in spin manipulation and selectivity of the spin-photon interface due to our observation of over 99% Rabi contrast in fluorescence readout, consistent with previous reports (*7*). This fidelity is greater than the SCC contrast (Fig. 4A) because of nonzero background counts and appreciable ionization during the readout window. We track the SCC end-to-end fidelity (*F*_SCC_) while sweeping the SCC pulse duration (Fig. 4C) and find that fidelity is maximized for a pulse duration of approximately 2 μs. We note that in Fig. 4B, there is significant population distribution above and below the *N* = 2 single-shot cutoff for both spin preparations, corresponding to a false-positive rate *p*_0*|*1_ = 27% and false-negative rate *p*_1*|*0_ = 11%, for *m_s_* = 0 and *m_s_* = +1, respectively (Supplementary Materials). This indicates that incomplete ionization of *m_s_* = 0 is the dominant source of infidelity in our SCC process, which we discuss below.

Although we increase the ionization laser power to maximize SCC contrast and fidelity, we observe that the SCC contrast (*C*_SCC_) unexpectedly saturates with power (Fig. 4D), limiting our single-shot readout fidelity. We attribute this saturation behavior to stimulated emission from the excited state induced by the 1151-nm ionization laser (*40*). Stimulated emission induces an excited–to–ground state transition of the defect, effectively increasing the spin-flip rate and decreasing the occupation time in the excited state. This results in a reduced chance of ionization via SCC before a spin-flip occurs and manifests as the saturating behavior seen in Fig. 4D. We model the dynamics of the SCC process using a set of differential equations to describe the various rates of ionization and spin-flips in our system (Supplementary Materials). Using the measured spin-agnostic ionization rate (Fig. 3D), the spin-flip rate, and the time evolution of Fig. 4A, our model predicts a maximum SCC fidelity of about 72%, which is consistent with our experimental findings. The reduction in the excited-state lifetime due to stimulated emission reduces the occupation of the excited state and lowers the ionization rate, which also explains the behavior seen in Fig. 3D. Last, by subtracting the spin-agnostic ionization rate (Fig. 3D) and the spin-flip rate from the SCC rate (Fig. 4A), we extract the stimulated emission rate. This rate is shown as a function of the ionization laser power in Fig. 4E (Supplementary Materials).

From our modeling, we find that the simple ratio of the ionization cross section from the excited state to the stimulated emission cross section (σ*_i_*/σ*_s_*) determines the resulting fidelity of SCC, where larger ratios are desirable. We directly calculate this metric with DFT, which indicates that the ratio is only optimal for a narrow energy window below the ZPL energy and above the energy of the first vibronic peak in the emission sideband (fig. S4, B and D). Our theoretical results also show that the cross sections and the ratio σ*_i_*/σ*_s_* do not change as a function of the light polarization when the incident light is parallel to the threefold rotation axis (*C*_3*v*_) of the defect (fig. S5, A and B). Unexpectedly, however, a large increase in the ratio σ*_i_*/σ*_s_* may be obtained by using polarized light perpendicular to the defect axis (Supplementary Materials). This suggests that a change in the ionization laser geometry and polarization may drastically increase the SCC fidelity. These investigations into the limitations of SCC due to stimulated emission through DFT and modeling create a set of guidelines and considerations for designing qubits and optimizing these types of spin-to-charge experiments.

### Extending coherence with dynamical decoupling

**Fig. 5.**
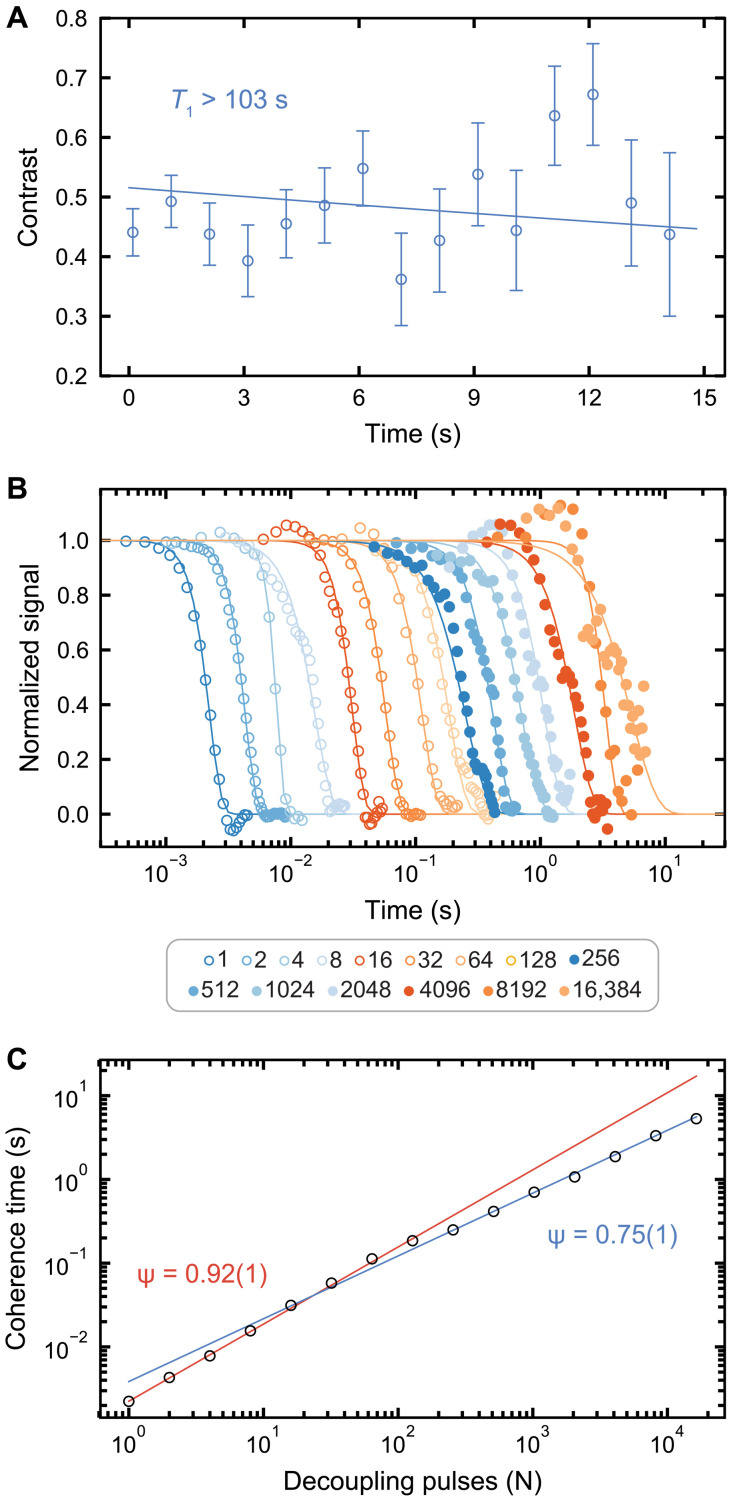
Ultralong spin coherence and lifetime for a single divacancy. (**A**) *T*_1_ spin relaxation time of divacancy using single-shot readout. Using a goodness of fit test with a 95% confidence interval, we estimate that *T*_1_ ≥ 103 s (Supplementary Materials). The fit (solid line) is for a *T*_1_ of 103 s. (**B**) *T*_2_ decay curves measured after applying dynamical decoupling pulses sequences of increasing pulse number, *N*. We avoid electron spin echo envelope modulation oscillations by enforcing pulse spacing requirements as in (*28*), eliminating sharp dips, and smoothing to find the coherence function envelope. The envelope is fit to a stretched exponential function ${\mathrm{Ae}}^{-{\left(\frac{t}{\tau }\right)}^{n}}$, where *n* is a stretch factor. (**C**) Extension of *T*_2_ coherence time with total decoupling pulse number. We fit in log space the low (blue) and high (red) pulse number regimes as *T*_2_ *~ N*^ψ^. All data are taken at *B* = 18 G and *T* = 5 K. All reported errors represent 1 SE from the fit, and all error bars represent 1 SD of the raw data.

Having demonstrated the ability to spin-selectively ionize the defect, we take advantage of the single-shot readout afforded by the SCC technique and perform measurements that reveal the exceptionally long spin coherence time of the defect’s spin state. We first perform *T*_1_ relaxation measurements using SCC readout (Fig. 5A). Although the charge state has appreciable decay on these time scales, we normalize our measurements such that we can extract the pure spin relaxation time. Despite no obvious spin *T*_1_ decay in Fig. 5A, we investigate the *T*_1_ time using a chi-square goodness of fit test and place a lower bound of 103 s on the *T*_1_ time with 95% confidence (Supplementary Materials). This minute-scale lower bound eliminates *T*_1_ spin relaxation as a concern for this system and is on par with the longest reported times for the NV^−^ center in diamond at equivalent temperatures (*28*).

In natural SiC, spin decoherence is dominated by magnetic fluctuations from flip-flop interactions between ^29^Si and ^13^C having *I* = ^1^/_2_ nuclear spins (*1*). The sample studied here was isotopically engineered to reduce the occurrence of nuclear flip-flops and extend the coherence time (Materials and Methods) (*7*). In this work, we further extend this spin coherence by applying dynamical decoupling sequences (fig. S8) (*24*). Figure 5B displays the coherence for sequences consisting of *N* = 1 to *N* = 16,384 pulses, measured with single-shot readout. With the combination of isotopic purification and dynamical decoupling, we measure a maximum *T*_2_ time of 5.3 ± 1.3 s, an improvement of over two orders of magnitude over the previously reported extended coherences in the divacancy system (*7*).

The dependence of coherence (*T*_2_) on pulse number (*N*) (Fig. 5C) can be modeled as *T*_2_ *~ N*^ψ^, where ψ varies with the frequency cutoff, shape, and roll-off behavior of sources of dephasing in the system (*41*). We find that the scaling of coherence for low pulse numbers has ψ *=* 0.92 ± 0.01 and ψ *=* 0.75 ± 0.01 at high pulse numbers (Fig. 5C). This may suggest that dephasing is dominated by two separate noise sources, each with differing associated frequency cutoffs that dominate in different regimes (*42*). The existence of these two competing noise sources is supported by previous work on the same isotopically purified sample that suggests that both a fast paramagnetic and slow nuclear spin bath play a role in dephasing (*7*), although certain broad Lorentzian baths may exhibit a similar ψ ~ 1 (*41*) to ψ ~ ^2^/_3_ crossover (*29*). Another possibility is that our experiments become dominated by control errors at high pulse number and do not protect the state as effectively, as we observe the contrast decreasing in this regime (fig. S9).

Even after over 10^4^ pulses, the measured coherence time does not saturate. Given the long *T*_1_ times, we expect coherence times greater than 5 s to be possible with even longer decoupling sequences (Materials and Methods). The combination of dynamical decoupling and isotopic purification in this work results in coherences that exceed state-of-the-art single spin measurements in other competing systems (*28*). These ultralong coherences offer many advantages for SiC-based quantum technologies. For example, in ac sensing protocols, long coherence times extend the phase accumulation period and increase sensitivity to weak signals. In addition, the reduction of memory storage errors that comes with long coherence times is vital for the development of quantum repeaters, which are a necessary component for future quantum networks.

## DISCUSSION

The all-optical SCC technique demonstrated here can be extended to other emitters where single-shot readout is needed (*43*–*45*), particularly in platforms where the photonic devices typically used to boost collection efficiency can degrade charge and optical qualities, or where detector or emitter quantum efficiency is low. In addition, coherence protection, combined with the high-fidelity readout provided by the SCC technique, brings the SiC material platform to the forefront for use in sensing and communication protocols that require both deterministic readout and quantum state preservation.

Looking forward, use of auxiliary microwave drives (*20*) to recover leakage of population from the qubit states, optimization of the ionization laser wavelength to maximize σ*_i_*/σ*_s_*, and further extension of the charge state stability via growth techniques may present pathways to improving the SCC technique. Our DFT calculations additionally suggest that a reduction in undesirable stimulated emission can be achieved by using polarized light with incidence orthogonal to the defect axis while still effectively ionizing the defect. We note, however, that the stimulated emission from defects is also of fundamental interest for future developments of lasers and new kinds of sensors (*46*). Furthermore, the charge itself may lead to new sensing opportunities (*47*), while the use of electrical depletion can reduce some of the remaining noise in the system to further increase coherence (*11*, *48*, *49*). Electron spin coherences in this platform could also be improved by further reducing magnetic noise through materials growth or by operating in decoherence protected subspaces with basal divacancy defects (*2*).

Integration of SCC with the optical tuning capabilities offered by SiC devices, such as p-i-n diodes (*11*), could be used to construct highly scalable, tunable SiC quantum nodes for future entanglement schemes. More generally, the SCC technique in SiC enables the translation of quantum spin-based information into charge-based information in a wafer-scale material with mature electronics technology. Thus, this work unlocks a new generation of devices where semiconductor structures such as metal oxide semiconductor field-effect transistors and avalanche photodiodes can be embedded with single spins to bridge the gap between quantum and classical electronic devices.

## MATERIALS AND METHODS

### Experimental details

Measurements are performed at *T* = 5 K in closed-cycle Montana Cryostat with a 0.85 numerical aperture near-infrared objective. All measurements are performed at low field of around ~18 G on a single *hh* divacancy in 4H-SiC. We use two TOPTICA DLC PRO lasers for narrow-line laser control of the defects. The 1151-nm ionization laser is a QPhotonics QFLD-1160-300S temperature tunable laser diode. PL is detected with a Quantum Opus superconducting nanowire single-photon detector. Photon counting and time tagging experiments use a PicoQuant PicoHarp 300 Time-Correlated Single-Photon Counting system. Charge initialization is achieved by applying a 705-nm light for 5 to 10 ms. For most experiments, the 705-nm laser power is ~200 nW at the sample. Spin initialization is achieved by pumping on the *E*_1,2_ transitions, while the *E_x_* transition serves as the cycling transition for readout and SCC. When performing SCC, to mitigate the effects of this nonselective ionization, we saturate the defect with resonant laser excitation to maximize the chances of desirable spin-selective ionization during the SCC step before a charge conversion error occurs. In addition, for subsequent experiments, we use the highest possible ionization laser power of 71 mW to increase the likelihood of ionization during the SCC step. This maximum power is also used to perform spin-agnostic ionization when preparing into the dark, ionized state in Fig. 2. Driving of transitions between the *m_s_* = 0 and *m_s_* = ±1 spin sublevels is performed using 1.357-GHz microwaves applied through Ti/Au striplines patterned on the sample surface. Microwave extinction and filtering are important parameters to increase coherence and lifetime. We use an 800-MHz high-pass filter after amplification and switch the microwave pulses on and off with a ZASWA-2-50DR+ both before and after amplification. Further extinction is achieved using the IQ modulation of the source, which also provides phase control of the microwave pulses. We use this phase control to perform the XY-8 pulse sequences for dynamical decoupling, which reduces sensitivity to pulse imperfections and drift. For all spin control experiments, we use π pulses with a length of approximately 1 to 2 μs. We stop our experiments at pulse number *N* = 16,384 for our dynamical decoupling experiments due to compounding pulse infidelity that reduces contrast and because our measured coherence approaches the charge lifetime for this defect. In Fig. 5B, the coherence function is normalized such that the contrast reduction and the finite charge lifetime do not affect the fitted coherences.

For the coherence measurements, two fiber-coupled Acousto-optic modulator (AOMs) (AA Opto-Electronic MT250-IR6-Fio-SMO) are used in series on the resonant lasers to achieve high extinction. Pulsing of the red charge reset tone (705 nm) and the 1151-nm ionization laser is achieved with direct modulation of the laser diode with a Thorlabs CLD1015 diode control unit. Extinction from this modulation is high, where we note that the finite charge lifetime is not affected by extra extinction of the 705-nm laser in the off state. All parameters in the text have errors reported at 1 SE.

### Isotopically purified sample

The sample consists of epitaxial 4H-SiC grown by chemical vapor deposition on a 4° off-axis n-type 4H-SiC. The layer thickness is ~90 μm and uses isotopically purified Si and C precursor gasses as in (*7*). Secondary ion mass spectroscopy reveals purities of 99.85% ^28^Si and 99.98% ^12^C. C-V measurements show slightly n-type behavior with a carrier concentration of 6 × 10^13^ cm^−3^. Single defects are created using a 1 × 10^13^ cm^−2^ dose of 2-MeV relativistic electrons. Subsequent annealing at 810°C in Ar gas results in isolated single VV^0^. We note that this slight n-type behavior causes VV^0^ to be unstable under illumination, where the negative charge states are favored. This is key, however, to our ability to ionize the VV^0^ effectively. The available carriers from these dopants provide the necessary charges to continually source and redistribute charges for these processes. SCC naturally requires charge unstable defects.

### Computational details

We carried out hybrid DFT calculations to determine the excitation energies of the VV^0^ defect and the charge transition energy to the VV^−^ defect. All calculations were performed using the dielectric-dependent hybrid functional (*50*) and the Quantum Espresso code (*51*). We used a 5 × 5 × 2 G-centered supercell, SG15 Optimized Norm-Conserving Vanderbilt (ONCV) pseudopotentials (*52*), and a plane-wave basis set with a kinetic energy cutoff of 80 rydberg. In the case of charged defects, we applied corrections to the total energy as derived in (*53*).

We computed the optical matrix elements pertaining to the ionization and stimulated emission cross sections using a G-centered supercell with 1296 atomic sites and the Perdew-Burke-Ernzerhof functional (*54*). Electron-phonon spectral functions were computed using the generating function approach within the displaced harmonic approximation (*40*, *55*, *56*). The phonon modes of the defective solid were obtained using a 5 × 5 × 2 supercell generated with the PHONOPY (*57*) package and extrapolated to larger sizes (16 × 16 × 5 supercell). Additional details are reported in the Supplementary Materials.

## Supplementary Materials

### This PDF file includes:

Supplementary Text Figs. S1 to S9 Table S1 References

## References

[1] H. Seo, A. L. Falk, P. V. Klimov, K. C. Miao, G. Galli, D. D. Awschalom, Quantum decoherence dynamics of divacancy spins in silicon carbide. Nat. Commun. 7, 12935 (2016). 27679936 10.1038/ncomms12935 PMC5056425

[2] K. C. Miao, J. P. Blanton, C. P. Anderson, A. Bourassa, A. L. Crook, G. Wolfowicz, H. Abe, T. Ohshima, D. D. Awschalom, Universal coherence protection in a solid-state spin qubit. Science 369, 1493–1497 (2020). 32792463 10.1126/science.abc5186

[3] D. J. Christle, A. L. Falk, P. Andrich, P. V. Klimov, J. U. Hassan, N. T. Son, E. Janzén, T. Ohshima, D. D. Awschalom, Isolated electron spins in silicon carbide with millisecond coherence times. Nat. Mater. 14, 160–163 (2015). 25437259 10.1038/nmat4144

[4] S. Kanai, F. J. Heremans, H. Seo, G. Wolfowicz, C. P. Anderson, S. E. Sullivan, G. Galli, D. D. Awschalom, H. Ohno, Generalized scaling of spin qubit coherence in over 12,000 host materials. arXiv:2102.02986 [quant-ph] (2021); http://arxiv.org/abs/2102.02986. 10.1073/pnas.2121808119 PMC9169712 35385350

[5] D. J. Christle, P. V. Klimov, C. F. de las Casas, K. Szász, V. Ivády, V. Jokubavicius, J. U. Hassan, M. Syväjärvi, W. F. Koehl, T. Ohshima, N. T. Son, E. Janzén, Á. Gali, D. D. Awschalom, Isolated spin qubits in SiC with a high-fidelity infrared spin-to-photon interface. Phys. Rev. X 7, 021046 (2017).

[6] K. C. Miao, A. Bourassa, C. P. Anderson, S. J. Whiteley, A. L. Crook, S. L. Bayliss, G. Wolfowicz, G. Thiering, P. Udvarhelyi, V. Ivády, H. Abe, T. Ohshima, Á. Gali, D. D. Awschalom, Electrically driven optical interferometry with spins in silicon carbide. Sci. Adv. 5, eaay0527 (2019). 31803839 10.1126/sciadv.aay0527 PMC6874486

[7] A. Bourassa, C. P. Anderson, K. C. Miao, M. Onizhuk, H. Ma, A. L. Crook, H. Abe, J. Ul-Hassan, T. Ohshima, N. T. Son, G. Galli, D. D. Awschalom, Entanglement and control of single nuclear spins in isotopically engineered silicon carbide. Nat. Mater. 19, 1319–1325 (2020). 32958880 10.1038/s41563-020-00802-6

[8] G. Wolfowicz, F. J. Heremans, C. P. Anderson, S. Kanai, H. Seo, A. Gali, G. Galli, D. D. Awschalom, Quantum guidelines for solid-state spin defects. Nat. Rev. Mater. 6, 906–925 (2021).

[9] A. L. Crook, C. P. Anderson, K. C. Miao, A. Bourassa, H. Lee, S. L. Bayliss, D. O. Bracher, X. Zhang, H. Abe, T. Ohshima, E. L. Hu, D. D. Awschalom, Purcell enhancement of a single silicon carbide color center with coherent spin control. Nano Lett. 20, 3427–3434 (2020). 32208710 10.1021/acs.nanolett.0c00339

[10] D. M. Lukin, C. Dory, M. A. Guidry, K. Y. Yang, S. D. Mishra, R. Trivedi, M. Radulaski, S. Sun, D. Vercruysse, G. H. Ahn, J. Vučković, 4H-silicon-carbide-on-insulator for integrated quantum and nonlinear photonics. Nat. Photonics 14, 330–334 (2020).

[11] C. P. Anderson, A. Bourassa, K. C. Miao, G. Wolfowicz, P. J. Mintun, A. L. Crook, H. Abe, J. U. Hassan, N. T. Son, T. Ohshima, D. D. Awschalom, Electrical and optical control of single spins integrated in scalable semiconductor devices. Science 366, 1225–1230 (2019). 31806809 10.1126/science.aax9406

[12] S. J. Whiteley, G. Wolfowicz, C. P. Anderson, A. Bourassa, H. Ma, M. Ye, G. Koolstra, K. J. Satzinger, M. V. Holt, F. J. Heremans, A. N. Cleland, D. I. Schuster, G. Galli, D. D. Awschalom, Spin–phonon interactions in silicon carbide addressed by Gaussian acoustics. Nat. Phys. 15, 490–495 (2019).

[13] S. J. Whiteley, F. J. Heremans, G. Wolfowicz, D. D. Awschalom, M. V. Holt, Correlating dynamic strain and photoluminescence of solid-state defects with stroboscopic x-ray diffraction microscopy. Nat. Commun. 10, 3386 (2019). 31358776 10.1038/s41467-019-11365-9 PMC6662806

[14] L. Robledo, L. Childress, H. Bernien, B. Hensen, P. F. A. Alkemade, R. Hanson, High-fidelity projective read-out of a solid-state spin quantum register. Nature 477, 574–578 (2011). 21937989 10.1038/nature10401

[15] M. Raha, S. Chen, C. M. Phenicie, S. Ourari, A. M. Dibos, J. D. Thompson, Optical quantum nondemolition measurement of a single rare earth ion qubit. Nat. Commun. 11, 1605 (2020). 32231204 10.1038/s41467-020-15138-7 PMC7105499

[16] N. T. Son, C. P. Anderson, A. Bourassa, K. C. Miao, C. Babin, M. Widmann, M. Niethammer, J. Ul Hassan, N. Morioka, I. G. Ivanov, F. Kaiser, J. Wrachtrup, D. D. Awschalom, Developing silicon carbide for quantum spintronics. Appl. Phys. Lett. 116, 190501 (2020).

[17] H. Bernien, B. Hensen, W. Pfaff, G. Koolstra, M. S. Blok, L. Robledo, T. H. Taminiau, M. Markham, D. J. Twitchen, L. Childress, R. Hanson, Heralded entanglement between solid-state qubits separated by three metres. Nature 497, 86–90 (2013). 23615617 10.1038/nature12016

[18] J. Cramer, N. Kalb, M. A. Rol, B. Hensen, M. S. Blok, M. Markham, D. J. Twitchen, R. Hanson, T. H. Taminiau, Repeated quantum error correction on a continuously encoded qubit by real-time feedback. Nat. Commun. 7, 11526 (2016). 27146630 10.1038/ncomms11526 PMC4858808

[19] D. M. Irber, F. Poggiali, F. Kong, M. Kieschnick, T. Lühmann, D. Kwiatkowski, J. Meijer, J. Du, F. Shi, F. Reinhard, Robust all-optical single-shot readout of nitrogen-vacancy centers in diamond. Nat. Commun. 12, 532 (2021). 33483515 10.1038/s41467-020-20755-3 PMC7822820

[20] Q. Zhang, Y. Guo, W. Ji, M. Wang, J. Yin, F. Kong, Y. Lin, C. Yin, F. Shi, Y. Wang, J. Du, High-fidelity single-shot readout of single electron spin in diamond with spin-to-charge conversion. Nat. Commun. 12, 1529 (2021). 33750779 10.1038/s41467-021-21781-5 PMC7943573

[21] J. R. Maze, P. L. Stanwix, J. S. Hodges, S. Hong, J. M. Taylor, P. Cappellaro, L. Jiang, M. V. G. Dutt, E. Togan, A. S. Zibrov, A. Yacoby, R. L. Walsworth, M. D. Lukin, Nanoscale magnetic sensing with an individual electronic spin in diamond. Nature 455, 644–647 (2008). 18833275 10.1038/nature07279

[22] J. M. Taylor, P. Cappellaro, L. Childress, L. Jiang, D. Budker, P. R. Hemmer, A. Yacoby, R. Walsworth, M. D. Lukin, High-sensitivity diamond magnetometer with nanoscale resolution. Nat. Phys. 4, 810–816 (2008).

[23] C. L. Degen, F. Reinhard, P. Cappellaro, Quantum sensing. Rev. Mod. Phys. 89, 035002 (2017).

[24] T. Gullion, D. B. Baker, M. S. Conradi, New, compensated Carr-Purcell sequences. J. Magn. Reson. 89, 479–484 (1990).

[25] H. Y. Carr, E. M. Purcell, Effects of diffusion on free precession in nuclear magnetic resonance experiments. Phys. Rev. 94, 630–638 (1954).

[26] J. T. Muhonen, J. P. Dehollain, A. Laucht, F. E. Hudson, R. Kalra, T. Sekiguchi, K. M. Itoh, D. N. Jamieson, J. C. McCallum, A. S. Dzurak, A. Morello, Storing quantum information for 30 seconds in a nanoelectronic device. Nat. Nanotechnol. 9, 986–991 (2014). 25305745 10.1038/nnano.2014.211

[27] A. M. Tyryshkin, S. Tojo, J. J. L. Morton, H. Riemann, N. V. Abrosimov, P. Becker, H.-J. Pohl, T. Schenkel, M. L. W. Thewalt, K. M. Itoh, S. A. Lyon, Electron spin coherence exceeding seconds in high-purity silicon. Nat. Mater. 11, 143–147 (2012). 10.1038/nmat3182 22138791

[28] M. H. Abobeih, J. Cramer, M. A. Bakker, N. Kalb, M. Markham, D. J. Twitchen, T. H. Taminiau, One-second coherence for a single electron spin coupled to a multi-qubit nuclear-spin environment. Nat. Commun. 9, 2552 (2018). 29959326 10.1038/s41467-018-04916-z PMC6026183

[29] N. Bar-Gill, L. M. Pham, A. Jarmola, D. Budker, R. L. Walsworth, Solid-state electronic spin coherence time approaching one second. Nat. Commun. 4, 1743 (2013). 23612284 10.1038/ncomms2771

[30] D. Simin, H. Kraus, A. Sperlich, T. Ohshima, G. V. Astakhov, V. Dyakonov, Locking of electron spin coherence above 20 ms in natural silicon carbide. Phys. Rev. B 95, 161201 (2017).

[31] N. T. Son, P. Carlsson, J. Ul Hassan, E. Janzén, T. Umeda, J. Isoya, A. Gali, M. Bockstedte, N. Morishita, T. Ohshima, H. Itoh, Divacancy in 4H-SiC. Phys. Rev. Lett. 96, 055501 (2006). 16486945 10.1103/PhysRevLett.96.055501

[32] P. Tamarat, N. B. Manson, J. P. Harrison, R. L. McMurtrie, A. Nizovtsev, C. Santori, R. G. Beausoleil, P. Neumann, T. Gaebel, F. Jelezko, P. Hemmer, J. Wrachtrup, Spin-flip and spin-conserving optical transitions of the nitrogen-vacancy centre in diamond. New J. Phys. 10, 045004 (2008).

[33] N. B. Manson, J. P. Harrison, M. J. Sellars, Nitrogen-vacancy center in diamond: Model of the electronic structure and associated dynamics. Phys. Rev. B 74, 104303 (2006).

[34] L. Gordon, A. Janotti, C. G. van de Walle, Defects as qubits in *3C*– and *4H*–SiC. Phys. Rev. B 92, 045208 (2015).

[35] G. Wolfowicz, C. P. Anderson, A. L. Yeats, S. J. Whiteley, J. Niklas, O. G. Poluektov, F. J. Heremans, D. D. Awschalom, Optical charge state control of spin defects in 4H-SiC. Nat. Commun. 8, 1876 (2017). 29192288 10.1038/s41467-017-01993-4 PMC5709515

[36] D. A. Golter, C. W. Lai, Optical switching of defect charge states in *4H*-SiC. Sci. Rep. 7, 13406 (2017). 29042675 10.1038/s41598-017-13813-2 PMC5645325

[37] B. Magnusson, N. T. Son, A. Csóré, A. Gällström, T. Ohshima, Á. Gali, I. G. Ivanov, Excitation properties of the divacancy in *4H*-SiC. Phys. Rev. B. 98, 195202 (2018).

[38] M. Bockstedte, F. Schütz, T. Garratt, V. Ivády, A. Gali, Ab initio description of highly correlated states in defects for realizing quantum bits. npj Quant. Mater. 3, 31 (2018).

[39] D. A. Hopper, H. J. Shulevitz, L. C. Bassett, Spin readout techniques of the nitrogen-vacancy center in diamond. Micromachines 9, 10.3390/mi9090437 (2018). 10.3390/mi9090437 PMC6187496 30424370

[40] L. Razinkovas, M. Maciaszek, F. Reinhard, M. W. Doherty, A. Alkauskas, Photoionization of negatively charged NV centers in diamond: Theory and ab initio calculations. Phys. Rev. B 104, 235301 (2021).

[41] M. J. Biercuk, H. Bluhm, Phenomenological study of decoherence in solid-state spin qubits due to nuclear spin diffusion. Phys. Rev. B 83, 235316 (2011).

[42] J. Medford, Ł. Cywiński, C. Barthel, C. M. Marcus, M. P. Hanson, A. C. Gossard, Scaling of dynamical decoupling for spin qubits. Phys. Rev. Lett. 108, 086802 (2012). 22463554 10.1103/PhysRevLett.108.086802

[43] G. Wolfowicz, C. P. Anderson, B. Diler, O. G. Poluektov, F. J. Heremans, D. D. Awschalom, Vanadium spin qubits as telecom quantum emitters in silicon carbide. Sci. Adv. 6, eaaz1192 (2020). 32426475 10.1126/sciadv.aaz1192 PMC7195180

[44] B. Diler, S. J. Whiteley, C. P. Anderson, G. Wolfowicz, M. E. Wesson, E. S. Bielejec, F. Joseph Heremans, D. D. Awschalom, Coherent control and high-fidelity readout of chromium ions in commercial silicon carbide. npj Quantum Inf. 6, 11 (2020).

[45] R. Nagy, M. Niethammer, M. Widmann, Y.-C. Chen, P. Udvarhelyi, C. Bonato, J. U. Hassan, R. Karhu, I. G. Ivanov, N. T. Son, J. R. Maze, T. Ohshima, Ö. O. Soykal, Á. Gali, S.-Y. Lee, F. Kaiser, J. Wrachtrup, High-fidelity spin and optical control of single silicon-vacancy centres in silicon carbide. Nat. Commun. 10, 1954 (2019). 31028260 10.1038/s41467-019-09873-9 PMC6486615

[46] J. Jeske, D. W. M. Lau, X. Vidal, L. P. McGuinness, P. Reineck, B. C. Johnson, M. W. Doherty, J. C. McCallum, S. Onoda, F. Jelezko, T. Ohshima, T. Volz, J. H. Cole, B. C. Gibson, A. D. Greentree, Stimulated emission from nitrogen-vacancy centres in diamond. Nat. Commun. 8, 14000 (2017). 28128228 10.1038/ncomms14000 PMC5290152

[47] G. Wolfowicz, C. P. Anderson, S. J. Whiteley, D. D. Awschalom, Heterodyne detection of radio-frequency electric fields using point defects in silicon carbide. Appl. Phys. Lett. 115, 043105 (2019).

[48] D. R. Candido, M. E. Flatté, Suppression of the optical linewidth and spin decoherence of a quantum spin center in a *p-i-n* diode. arXiv:2008.13289 [cond-mat.mes-hall] (2020); https://arxiv.org/abs/2008.13289.

[49] K. Ghosh, H. Ma, M. Onizhuk, V. Gavini, G. Galli, Spin–spin interactions in defects in solids from mixed all-electron and pseudopotential first-principles calculations. npj Comput. Mater. 7, 123 (2021).

[50] J. H. Skone, M. Govoni, G. Galli, Self-consistent hybrid functional for condensed systems. Phys. Rev. B 89, 195112 (2014).

[51] P. Giannozzi, S. Baroni, N. Bonini, M. Calandra, R. Car, C. Cavazzoni, D. Ceresoli, G. L. Chiarotti, M. Cococcioni, I. Dabo, A. D. Corso, S. de Gironcoli, S. Fabris, G. Fratesi, R. Gebauer, U. Gerstmann, C. Gougoussis, A. Kokalj, M. Lazzeri, L. Martin-Samos, N. Marzari, F. Mauri, R. Mazzarello, S. Paolini, A. Pasquarello, L. Paulatto, C. Sbraccia, S. Scandolo, G. Sclauzero, A. P. Seitsonen, A. Smogunov, P. Umari, R. M. Wentzcovitch, QUANTUM ESPRESSO: A modular and open-source software project for quantum simulations of materials. J. Phys. Condens. Matter 21, 395502 (2009). 21832390 10.1088/0953-8984/21/39/395502

[52] M. Schlipf, F. Gygi, Optimization algorithm for the generation of ONCV pseudopotentials. Comput. Phys. Commun. 196, 36–44 (2015).

[53] C. Freysoldt, J. Neugebauer, C. G. van de Walle, Fully *Ab Initio* finite-size corrections for charged-defect supercell calculations. Phys. Rev. Lett. 102, 016402 (2009). 19257218 10.1103/PhysRevLett.102.016402

[54] J. P. Perdew, K. Burke, M. Ernzerhof, Generalized gradient approximation made simple. Phys. Rev. Lett. 77, 3865–3868 (1996). 10062328 10.1103/PhysRevLett.77.3865

[55] Y. Jin, M. Govoni, G. Wolfowicz, S. E. Sullivan, F. J. Heremans, D. D. Awschalom, G. Galli, Photoluminescence spectra of point defects in semiconductors: Validation of first-principles calculations. Phys. Rev. Materials. 5, 84603 (2021).

[56] L. Razinkovas, M. W. Doherty, N. B. Manson, C. G. de Walle, A. Alkauskas, Vibrational and vibronic structure of isolated point defects: The nitrogen-vacancy center in diamond. Phys. Rev. B. 104, 45303 (2021).

[57] A. Togo, I. Tanaka, First principles phonon calculations in materials science. Scr. Mater. 108, 1–5 (2015).

[58] H.-Y. Chen, M. Palummo, D. Sangalli, M. Bernardi, Theory and ab initio computation of the anisotropic light emission in monolayer transition metal dichalcogenides. Nano Lett. 18, 3839–3843 (2018). 29737164 10.1021/acs.nanolett.8b01114

[59] P. Giannozzi, O. Andreussi, T. Brumme, O. Bunau, M. B. Nardelli, M. Calandra, R. Car, C. Cavazzoni, D. Ceresoli, M. Cococcioni, N. Colonna, I. Carnimeo, A. D. Corso, S. de Gironcoli, P. Delugas, R. A. DiStasio, A. Ferretti, A. Floris, G. Fratesi, G. Fugallo, R. Gebauer, U. Gerstmann, F. Giustino, T. Gorni, J. Jia, M. Kawamura, H.-Y. Ko, A. Kokalj, E. Küçükbenli, M. Lazzeri, M. Marsili, N. Marzari, F. Mauri, N. L. Nguyen, H.-V. Nguyen, A. Otero-de-la-Roza, L. Paulatto, S. Poncé, D. Rocca, R. Sabatini, B. Santra, M. Schlipf, A. P. Seitsonen, A. Smogunov, I. Timrov, T. Thonhauser, P. Umari, N. Vast, X. Wu, S. Baroni, Advanced capabilities for materials modelling with Quantum ESPRESSO. J. Phys. Condens. Matter 29, 465901 (2017). 29064822 10.1088/1361-648X/aa8f79

[60] P. Giannozzi, O. Baseggio, P. Bonfà, D. Brunato, R. Car, I. Carnimeo, C. Cavazzoni, S. de Gironcoli, P. Delugas, F. F. Ruffino, A. Ferretti, N. Marzari, I. Timrov, A. Urru, S. Baroni, Quantum ESPRESSO toward the exascale. J. Chem. Phys. 152, 154105 (2020). 32321275 10.1063/5.0005082

[61] L. Patrick, W. J. Choyke, Static dielectric constant of SiC. Phys. Rev. B. 2, 15 (1970).

[62] Y. Goldberg, M. Levinshtein, S. Rumyantsev, *Properties of Advanced Semiconductor Materials: GaN, AIN, InN, BN, SiC, SiGe* (John Wiley & Sons, 2001).

[63] M. Govoni, G. Galli, Large scale GW calculations. J. Chem. Theory Comput. 11, 2680–2696 (2015). 26575564 10.1021/ct500958p

