# Supplementary material for: Five-second coherence of a single spin with single-shot readout in silicon carbide
Source: Sci Adv. 2022 Feb 2;8(5):eabm5912. doi: 10.1126/sciadv.abm5912 (PMC8809532; doi:10.1126/sciadv.abm5912)
Supplement: Supplementary file 1 — Supplementary Text Figs. S1 to S9 Table S1 References [file sciadv.abm5912_sm.pdf]

Science Advances
AAAS

Supplementary Materials for

Five-second coherence of a single spin with single-shot readout in silicon carbide

Christopher P. Anderson, Elena O. Glen, Cyrus Zeledon, Alexandre Bourassa, Yu Jin, Yizhi Zhu, Christian Vorwerk, Alexander L. Crook, Hiroshi Abe, Jawad Ul-Hassan, Takeshi Ohshima, Nguyen T. Son, Giulia Galli, David D. Awschalom*

*Corresponding author. Email: awsch@uchicago.edu

Published 2 February 2022, *Sci. Adv.* **8**, eabm5912 (2022)
DOI: 10.1126/sciadv.abm5912

This PDF file includes:

Supplementary Text
Figs. S1 to S9
Table S1
References

Supplementary Text

I. Charge lifetime calculation

For the charge lifetime measurement, we model the decay in the PL signal as:

$$A * \exp\left(-\frac{t}{\tau_{ch}}\right) + y_0 \qquad (Eq. S1)$$

where A is the PL rate, y_0 is the background laser scatter and dark count rate, and τ_{ch} is the charge lifetime. From our data we find $\tau_{ch} = 6.98 \pm 0.89$ s, as shown in the fit displayed in Fig. 2A.

II. Mean photon number for photon number distribution calculation

For a normalized, discrete photon number distribution m_j, the mean value μ of the distribution is calculated as:

$$\mu = \sum_{j=0}^{len(m_j)} j * m_j \qquad (Eq. S2)$$

where j is the photon number. The error of the mean is taken to be $\sqrt{\mu}$.

III. Projected photons per shot calculation for charge readout

The PL signal decays over a charge readout window as:

$$A * \exp\left(-\frac{t}{\tau}\right) \qquad (Eq. S3)$$

where A is the PL rate with units of photon/second, and τ is the decay constant in seconds. From this, we calculate the projected photons per shot for a given power as $A * \tau$ which is the integral of the exponential decay to infinity. We use this expression to find the dependence of the photons per shot n_{shot} on resonant laser power p. It takes the form:

$$n_{shot}(p) = A(p) * \tau_{ion}(p) \qquad (Eq. S4)$$

The ionization rate, $1/\tau_{ion}$, takes the form:

$$\frac{1}{\tau_{ion}} = \frac{1}{t_0} + \frac{p * b}{1 + \frac{p_{sat}}{p}} \qquad (Eq. S5)$$

where t_0 is the charge lifetime, p_{sat} is the saturation power, and b is the ionization rate at saturation. This equation represents both the intrinsic charge lifetime decay and the two-photon

ionization from a saturable two-level system. Additionally, *A (p)* is the power-dependent PL rate which saturates with power:

$$A(p) = \frac{c}{1 + \frac{p_{sat}}{p}} \qquad (Eq. S6)$$

where *c* is the photoluminescence rate at saturation and p_{sat} is the saturation power.
To find $n_{shot}(p)$, we use Eq. S5 and Eq. S6 to evaluate $A * \tau$ and find that:

$$n_{shot}(p) = \frac{c * t_0 * p}{p + p_0 + b * t * p^2} \qquad (Eq. S7)$$

We use Eq. S7 to calculate the projected photons per shot as displayed by the fit in Fig. 2C.

IV. Charge repump/reinitialization rate calculation

As the duration of the 705 nm charge reset pulse increases, the PL signal for the subsequent charge readout steps grows as:

$$A * \left(1 - \exp\left(-\frac{t - t_0}{\tau_r}\right)\right) \qquad (Eq. S8)$$

where A is the PL rate, t_0 is a time offset, and τ_r is the characteristic charge initialization time. We define the charge reset rate to be $1/\tau_r$.

V. Charge readout fidelity and end-to-end fidelity of SCC calculation

We take the definition of fidelity from Ref. (*39*) as:

$$F = 1 - \frac{1}{2}\left(1 - p_{0|0} + p_{0|1}\right) = \frac{1}{2}\left(p_{0|0} + p_{1|1}\right) \qquad (Eq. S9)$$

where $p_{0|0}$ is the normalized true positive probability, $p_{0|1}$ is the normalized false positive probability, $p_{1|1}$ is the normalized true negative probability and $(1 - p_{0|1}) = p_{1|1}$. Using Eq. S9, for a single shot cutoff of *n* photons, the single shot charge readout fidelity is calculated as:

$$F_{charge} = \frac{1}{2}\left(\frac{\sum_0^n p_d}{\sum_0^\infty p_d} + \frac{\sum_n^\infty p_b}{\sum_0^\infty p_b}\right) \qquad (Eq. S10)$$

Where p_d and p_b are the unnormalized photon distributions for the prepared dark and bright states, respectively. Similarly, for a single shot cutoff of *n* photons, the end-to-end SCC fidelity is calculated as:

$$F_{SCC} = \frac{1}{2}\left(\frac{\sum_0^n p_{+1}}{\sum_0^\infty p_{+1}} + \frac{\sum_n^\infty p_0}{\sum_0^\infty p_0}\right) \qquad (Eq. S11)$$

where p_0 and p_{+1} are the unnormalized single shot photon number distributions after preparation into $m_s = 0$ and $m_s = +1$, respectively. Eq. S11 is used to calculate the data displayed in Fig. 4C. In all experiments, the cutoff *n* is chosen to maximize *F*.

VI. Fitting of SCC traces and SCC contrast calculation

We fit the signal of the charge readout after varying the SCC duration as an exponential decay:

$$A_i e^{-\frac{t}{\tau_i}} + y_i \qquad (Eq.S12)$$

where A_i and y_i are PL rates, and τ_i is the decay time constant. We calculate the contrast C_{SCC} in the charge readout between $m_s = 0$ and $m_s = +1$, denoted by the subscripts 0 and +1, after a given SCC pulse duration t_{ion} as:

$$C_{SCC} = \frac{\left(A_{+1} e^{-\frac{t_{ion}}{\tau_{+1}}} + y_{+1}\right) - \left(A_0 e^{-\frac{t_{ion}}{\tau_0}} + y_0\right)}{\frac{1}{2}\left(A_{+1} + y_{+1} + A_0 + y_0\right)} \qquad (Eq.S13)$$

The fits in Eq. S13 are used to calculate the contrast to better eliminate noise and to allow for better error estimation.

VII. Chi-squared goodness of fit test to predict T1 longitudinal lifetime

To place a lower bound on the T_1 time of the defect we perform a χ^2 test for goodness of fit by producing a χ^2-distribution comparing our data to equations of the form:

$$A * \exp\left(-\frac{t}{T_{1,test}}\right) \qquad (Eq.S14)$$

We first fit the PL rate, *A*, to our collected data for a given $T_{1,test}$ value. We then calculate the χ^2 value of our test T_1 decay curve to the data. The χ^2 value is calculated as:

$$\chi^2 = \sum_{i=1}^{k} \left(\frac{X_i - \mu_i}{\sigma_i}\right)^2 \qquad (Eq.S15)$$

where X_i is the measured value, μ_i is the predicted value from the generated decay curve, σ_i is the standard deviation of the measured value. Here, there are $k = 15$ data points that each correspond to a different time t_i.

We use a χ^2goodness of fit test to determine the lower bound on the T_1 value. For a right-handed test with a confidence level of 95% and 13 degrees of freedom (corresponding to the k=15 data points and 2 fit parameters), the critical chi-squared value is 22.362. We find that all chi-squared distributions generated for $T_{1,test} < 103.3$ seconds exceed this critical value. Thus, we place a lower bound estimate of 103.3 seconds on the T_1 time with 95% confidence.

VIII. Modelling the SCC process:

To model the dynamics of the SCC process, we create a set of differential equations describing the rates of ionization and spin-flips in the system. We assume the orbital dynamics of the defect are fast and average over the photon absorption and emission events, treating the population in the excited state (P_{ES}) generally in the form of a saturation curve. The saturation curve is generally in the form:

$$P_{ES}(p) = \frac{A}{1 + p_0/p} = \frac{A}{1 + \tau_{absorb}/\tau_{emit}} = \frac{A}{1 + R_{emit}/R_{absorb}} \qquad (Eq.S16)$$

with p_0 representing the power by which a photon is absorbed on average once every optical lifetime ("saturation"). As shown above, the factor p_0/p can also be understood as the ratio of time for absorption and emission, or equivalently the ratio of emission to absorption rates. Because the spin-flip process is due to a set branching ratio per occupation of the excited state, the spin-flip rate (R_{sf}) also follows a power-dependent saturation curve with respect to the resonant power p_{res}:

$$R_{sf}(p_{res}) = \frac{R_{sat}}{1 + p_0/p_{res}} \qquad (Eq.S17)$$

with R_{sat} as the saturated spin-flip rate. On the other hand, the ionization rate (R_I) from the excited state is also related to the population in the excited state times a one-photon power dependent rate ionizing from that state. We denote the ionization laser power p_i, where assuming the resonant-only two-photon ionization rate is low:

$$R_I(p_{res}, p_i) = \frac{a\, p_i}{1 + p_0/p_{res}} \qquad (Eq.S18)$$

where a is the linear ionization rate per unit power. We note here that the rate from the excited state is actually *2a*, given that the excited state is only populated half of the time when saturated. With our resonant laser only pumping the highly cycling E_x transition, the spin flips deplete the $m_s = 0$ state and populate the $m_s = \pm 1$ state. This results in the following equations for the populations in the bright $m_s = 0$ (B_0), bright $m_s = \pm 1$ (B_1), and dark populations (D):

$$\frac{dB_0}{dt} = -R_I(p_{res}, p_i)B_0 - R_{sf}(p_{res})B_0 \qquad (Eq.S19)$$

$$\frac{dB_1}{dt} = +R_{sf}(p_{res})B_0 \qquad (Eq.S20)$$

$$\frac{dD}{dt} = +R_I(p_{res}, p_i)B_0 \qquad (Eq.S21)$$

We can solve Eq. S19-S21 assuming that the system is either initialized into $m_s = 0$ (B_0), where $B_0(0) = 1$, or $m_s = \pm 1$ where $B_1(0) = 1$. Upon charge readout, we only see if the defect is bright or dark, measuring $B(t) = B_0(t) + B_1(t)$ with $B(t) + D(t) = 1$. The analytic form of the time dependence during the SCC process upon solving becomes:

$$B(t) = \frac{R_{sf} + R_I e^{-(R_{sf}+R_I)t}}{R_{sf} + R_I} \qquad (Eq. S22)$$

where the exponential term has a rate $R_{SCC} = R_{sf} + R_I$
We use Eq. S22 to model the effect of the balance of ionization rate and spin-flip rates on the final maximal SCC contrast $C = max\big(1 - B(t)\big)$. In this case, this occurs when $t \to \infty$:

$$C(p_i) = \frac{R_I}{R_{sf} + R_I} = \frac{1}{1 + \frac{R_{sat}}{ap_i}} \qquad (Eq. S23)$$

Which we can recognize as a saturation curve with respect to the ionization laser power, in which the relevant rate and timescale is set by the saturation spin flip rate R_{sat} and ap_i is the ionization rate when the defect is saturated with the resonant laser. In this case, near-unity contrast can be achieved simply by having sufficient ionization laser power. In this simplified model, which does not take into account dynamics in the readout or experimental imperfections, the contrast is the same as the SCC fidelity (*C*=*F*).

i) Including the effect of stimulated emission

In our experiment, we hypothesize that the reason that contrast does not approach unity even when the ionization laser power is increased is due to stimulated emission from the defect excited state induced by the ionization laser (1151 nm). This laser is too high in wavelength to be absorbed by the defect, but does overlap with the emission sideband. A sufficiently strong pump can stimulate the defect to emit at the particular ionization laser frequency. The result of stimulated emission is a reduction in the effective excited state lifetime. This results in two changes to the relevant rates. The first effect is that it modifies the saturation behavior of the defect. A power that was saturating the defect without stimulated emission may be too weak and will not saturate the defect when the lifetime becomes effectively shortened. We model this effect by a modification of $p_0 \to p_0 + \alpha p_i$, which represents a linear increase in the emission rate from the excited state. Additionally, the "saturated" spin flip rate will be modified due to the reduced excited state lifetime, which is linear in power $R_{sat} \to R_{sat} + \beta p_i$, where p_i the ionization laser power. Combined, we have for the spin-flip rate:

$$R_{sf,stim}(p_{res}, p_i) = \frac{R_{sat} + \beta p_i}{1 + (p_0 + \alpha p_i)/p_{res}} \qquad (Eq. S24)$$

The second effect is a change of the ionization rate. This is purely due to the modification of the saturation behavior.

$$R_{I,stim}(p_{res}, p_i) = \frac{a\, p_i}{1 + (p_0 + \alpha p_i)/p_{res}} \qquad (Eq. S25)$$

We note that this unexpected saturation is shown in experiment in Fig. 3D. The rate equations are the same as described previously, just with the modified power dependence and interplay

between p_i and p_{res}. The SCC contrast under the effect of stimulated emission has the same solution as before, with modified R_I and R_{sf}.
Critically, the time constant on the exponential term is still $R_{sf,stim} + R_{I,stim}$ and the contrast at $\tau \to \infty$ is now:

$$C(p_i) = \frac{1}{1 + \frac{R_{sat}}{ap_i} + \frac{\beta}{a}} \qquad (Eq.S26)$$

The maximal contrast is still achieved where $p_i \to \infty$, but is non-unity:

$$C_{max} = \frac{1}{1 + \frac{\beta}{a}} \qquad (Eq.S27)$$

We see that the maximal contrast C_{max} is limited by the ratio of the stimulated emission-induced spin-flip increase from a lifetime reduction (β) to the ionization rate from the ionization laser (a). We note that the maximal contrast does not depend on the effect of the saturation behavior modification (the factor α). Generally, from the form of $C(p_i)$, the contrast increases with p_i, but saturates to the non-unity value C_{max}. We use this function to fit Fig. 4D.
We can also calculate the time evolution in the case where we excite both on the E_x and $E_{1,2}$ lines, constituting a spin-independent mechanism to access the excited state with our resonant lasers. In this case, the ionization is spin-agnostic and the rate equations:

$$\frac{dB}{dt} = -R_{I,stim}(p_{res}, p_i)B \qquad (Eq.S28)$$

$$\frac{dD}{dt} = +R_{I,stim}(p_{res}, p_i)B \qquad (Eq.S29)$$

and therefore, when prepared in the bright state:

$$B(t) = e^{-R_{I,stim}t} \qquad (Eq.S30)$$

ii) Calculating the stimulated emission contribution

In experiment, we can measure the saturated spin flip rate R_{sat} (Fig. S1). We can also perform spin agnostic-ionization to measure the rate of decay $R_{I,stim}$. Finally, we can use the decay of the population in the spin dependent SCC experiment which has a rate $R_{sf,stim} + R_{I,stim}$ as mentioned before. We note that the SCC curve has contributions from both the spin flip rate, the ionization rate, and a modification from the stimulated emission rate. Our goal is to elucidate the contribution from stimulated emission by itself. Without considering the contribution from stimulated emission, we can take the results from our three experiments and calculate,

$R_{stim} = R_{SCC} - R_{spin-agnostic} - R_{sat} = (R_I + R_{sf}) - R_I - R_{sat} = R_{sf} - R_{sat}$. In our experiment, we operate where the defect is saturated $p_{res} \gg p_0$ and thus $R_{sf} \approx R_{sat}$. Therefore, we expect in the absence of stimulated emission, $R_{SCC} - R_{spin-agnostic} - R_{sat} = 0$. Anything non-zero in our experiment represents the effect of the modifications from stimulated emission. So, we have the expression for the "missing rate", R_{stim}:

$$R_{stim} = R_{sf,stim}(p_{res}, p_i) - R_{sat} \qquad (Eq. S31)$$

which we re-write:

$$R_{stim} = \frac{\beta p_i}{1 + (p_0 + \alpha p_i)/p_{res}} + \left(-1 + \frac{1}{1 + (p_0 + \alpha p_i)/p_{res}}\right) R_{sat} \qquad (Eq. S32)$$

If once again we assume $p_{res} \gg p_0 + \alpha p_i$, where the saturation reduction effect is minimized then we see:

$$R_{stim} = \beta p_i \qquad (Eq. S33)$$

which cancels all terms except the effect of stimulated emission. The assumption $p_{res} \gg p_0 + \alpha p_i$ is validated by the non-saturating behavior in Fig. 4E, which plots this missing rate. Our calculation then allows us to extract β, the stimulated emission effect on the spin-flip rate per unit power. In experiment we measure ~13 MHz/W (Fig. 4E). In practice, the actual stimulated emission rate is 2β, once again due to the 50% occupation of the excited state at saturation. We can also directly measure α, the spin agnostic ionization rate per unit power as the linear slope of Fig. 3D. In experiment, the behavior is slightly saturating, with a fitted value using the low power data for α of ~35 MHz/W. Fig. 3D is fit well to the saturation behavior of $R_{I,stim}(p_{res}, p_i)$, further evidence of the role stimulated emission plays. From these two measurements, the resulting ratio $\frac{\beta}{\alpha}$ is ~0.37. We note that from our rate equation model, we therefore expect the maximal contrast/fidelity from this basic model to be approximately $C_{max} = \frac{1}{1+\frac{\beta}{\alpha}}$ ~ 72%, surprisingly consistent with our results given just the measurements of α, β. The matching of the power dependencies (unexpected saturation), the presence of this "missing rate" and the estimation of our contrast by just measuring two relevant rates establishes that stimulated emission plays a dominant role in the SCC process. From the fits of the data, we can further estimate $\frac{\alpha}{p_{res}}$ ~9 at with p_{res}~14 μW. Similarly, we can confirm the ratio of $\frac{p_0}{p_{res}}$ which we measure to be ~0.27 in experiment, consistent with the fit from our modeling ~0.18.

IX. Density functional theory (DFT) calculations

i) Theory and methods

a. Energetics

We computed the energy of the ionization threshold ($\boldsymbol{E_{q+1/q}^{VBM}}$) from VV^0 to VV^- using Kohn-Sham density functional theory (DFT):

$$\boldsymbol{E^{VBM}_{q+1/q} = E^{q}_{tot} - E^{q+1}_{tot} + \Delta V(q) - \Delta V(q+1) - \varepsilon(VBM)} \qquad (Eq.S34)$$

where q= -1 for VV^- and q+1=0 for VV^0 , E_{tot} is the total energy of the supercell containing the defect, ΔV is a correction term to the total energy as derived by Freysoldt et al. in the case of charged defects (*53*), and $\varepsilon(VBM)$ is the position of valence band maximum (VBM) as determined by the highest occupied Kohn-Sham eigenvalue.

b. Cross sections

The cross section of the stimulated emission as a function of the photon energy $\hbar\omega$ and temperature T is given by (*40*):

$$\sigma_\mathrm{s}(\hbar\omega, T) = \frac{4\pi^2\alpha}{n}\hbar\omega r_s^2 A_\mathrm{s}(\hbar\omega - E_{ZPL}, T) \qquad (Eq.S35)$$

Here α is the fine-structure constant, n is the refractive index, r_s^2 is the square of the optical matrix element for the transition ^3E to $^3A_2$ of VV^0 at a given photon polarization, and E_{ZPL} is the energy of the zero-phonon line (ZPL). $A_\mathrm{s}(\hbar\omega, T)$ is the electron-phonon spectral function for stimulated emission, which is the same as that of the spontaneous emission (photoluminescence) (*55*, *56*):

$$A_\mathrm{s}(\hbar\omega, T) = \sum_a \sum_b P_{e_a}(T)\left|\langle \Theta_{e_a}|\Theta_{g_b}\rangle\right|^2 \delta\left(\hbar\omega - E_{e_a} + E_{g_b}\right) \qquad (Eq.S36)$$

Here Θ_{e_a} (Θ_{g_b}) is the ath (b^{th}) nuclear wave function of the system in the excited state ^3E (ground state $^3A_2$) with vibrational energy E_{e_a} (E_{g_b}), and $P_{e_a}(T)$ is the distribution function of the vibrational energies at finite temperature in the excited state. We compute the spectral function by using the displaced harmonic oscillator approximation and the generating function approach (*55*).

Similarly, the ionization cross section is given by:

$$\sigma_\mathrm{i}(\hbar\omega, T) = \frac{4\pi^2\alpha}{n}\hbar\omega \sum_j r_{i,j}^2 A_\mathrm{i}\left(\hbar\omega - E_{i,j}, T\right) \qquad (Eq.S37)$$

Here $r_{i,j}^2$ is the square of the optical matrix element between the initial state (^3E state of VV^0), and all possible final states (^2E state of VV^- with a hole in the valence band) for a given photon polarization direction. The summation over j includes all valence states of the defective system. $E_{i,j}$ is the energy difference between the initial and final states. $A_\mathrm{i}(\hbar\omega, T)$ is the electron-phonon spectral function for ionization, which can be computed in the same way as $A_\mathrm{s}(\hbar\omega, T)$. We computed the ionization cross section as a convolution (*40*):

$$\sigma_\mathrm{i}(\hbar\omega, T) = \hbar\omega \int_{-\infty}^{\infty} \frac{1}{\hbar\omega'} \tilde{\sigma}_\mathrm{i}(\hbar\omega') A_\mathrm{i}(\hbar\omega - \hbar\omega') d(\hbar\omega') \qquad (Eq.S38)$$

where

$$\tilde{\sigma}_{\mathrm{i}}(\hbar\omega) = \frac{4\pi^2\alpha}{n}\hbar\omega\sum_{j} r_{i,j}^2\delta\left(\hbar\omega - E_{\mathrm{i},j}\right) \qquad (Eq.S39)$$

Here, we replace the δ function with a Gaussian function, since in our calculations we utilize a finite number of k-points to sample the supercell Brillouin zone.

c. Optical matrix elements

Optical matrix elements are key ingredients for the accurate description of both the stimulated-emission and ionization cross sections. Here we compute optical matrix elements assuming that the wavefunctions of the single-particle orbitals of the defective crystal are the same irrespective of the orbital occupation. Hence, the optical matrix elements between two many-electron wavefunctions can be computed from the matrix elements between single particle orbitals (*40*). For example, the optical matrix elements entering the stimulated emission cross section for the $m_s = 1$ sublevel of the ^3E and the $^3A_2$ state of VV^0 are given by:

$$\begin{aligned}\boldsymbol{r}_s &= \left\langle {}^3E_{x/y}, m_s = 1\right| \textstyle\sum_e \boldsymbol{r}_e \left| {}^3A_2, m_s = 1\right\rangle \\ &= \left\langle \bar{a}_1\bar{e}_{x/y}\right| \textstyle\sum_e \boldsymbol{r}_e \left|\bar{e}_{y/x}\bar{e}_{x/y}\right\rangle = \left\langle \bar{a}_1\middle|\boldsymbol{r}\middle|\bar{e}_{y/x}\right\rangle \qquad (Eq.S40)\end{aligned}$$

where we use the hole representation for many-electron wavefunctions. A similar expression is obtained for the $m_s = 0$ and $m_s = -1$ sublevels, where we assume that the spin-up and spin-down orbitals are identical.

The ionization optical matrix elements are calculated in a way similar to those of the stimulated emission. The optical matrix element from the $m_s = 1$ and $m_s = -1$ sublevels of the ^3E state of VV^0 into the $m_s = \frac{1}{2}$ and $m_s = -\frac{1}{2}$ sublevels of the ^2E state of VV^- with a hole in the valence band are given by:

$$\begin{aligned}\boldsymbol{r}_{i,j} &= \left\langle {}^3E_{x/y}, m_s = 1\middle| \textstyle\sum_e \boldsymbol{r}_e \middle| \left({}^2E_{x/y}, m_s = \frac{1}{2}\right) + \bar{\phi}_j\right\rangle \\ &= \left\langle \bar{a}_1\bar{e}_{x/y}\right| \textstyle\sum_e \boldsymbol{r}_e \left|\bar{e}_{x/y}\bar{\phi}_j\right\rangle = \left\langle \bar{a}_1\middle|\boldsymbol{r}\middle|\bar{\phi}_j\right\rangle \qquad (Eq.S41)\end{aligned}$$

$$\begin{aligned}\boldsymbol{r}_{i,j} &= \left\langle {}^3E_{x/y}, m_s = -1\middle| \textstyle\sum_e \boldsymbol{r}_e \middle| \left({}^2E_{x/y}, m_s = -\frac{1}{2}\right) + \phi_j\right\rangle \\ &= \left\langle a_1 e_{x/y}\right| \textstyle\sum_e \boldsymbol{r}_e \left|e_{x/y}\phi_j\right\rangle = \left\langle a_1\middle|\boldsymbol{r}\middle|\phi_j\right\rangle \qquad (Eq.S42)\end{aligned}$$

In an analogous fashion, the optical matrix element from the $m_s = 0$ sublevel of the ^3E state of VV^0 into the $m_s = \frac{1}{2}$ and $m_s = -\frac{1}{2}$ sublevels of the ^2E state of VV^- with a hole in the valence band are given by:

$$\begin{aligned}\boldsymbol{r}_{i,j} &= \left\langle {}^3E_{x/y}, m_s = 0\middle| \textstyle\sum_e \boldsymbol{r}_e \middle| \left({}^2E_{x/y}, m_s = \frac{1}{2}\right) + \phi_j\right\rangle \\ &= \frac{1}{\sqrt{2}}\left\langle a_1\bar{e}_{x/y}\right| \textstyle\sum_e \boldsymbol{r}_e \left|\bar{e}_{x/y}\phi_j\right\rangle + \frac{1}{\sqrt{2}}\left\langle \bar{a}_1 e_{x/y}\right| \textstyle\sum_e \boldsymbol{r}_e \left|\bar{e}_{x/y}\phi_j\right\rangle = \frac{1}{\sqrt{2}}\left\langle a_1\middle|\boldsymbol{r}\middle|\phi_j\right\rangle \qquad (Eq.S43)\end{aligned}$$

$$\boldsymbol{r}_{i,j} = \left\langle {}^{3}E_{x/y}, m_s = 0 \middle| \sum_e \boldsymbol{r}_e \middle| \left({}^{2}E_{x/y}, m_s = -\frac{1}{2}\right) + \bar{\phi}_j \right\rangle$$
$$= \frac{1}{\sqrt{2}} \langle a_1 \bar{e}_{x/y} | \sum_e \boldsymbol{r}_e | e_{x/y} \bar{\phi}_j \rangle + \frac{1}{\sqrt{2}} \langle \bar{a}_1 e_{x/y} | \sum_e \boldsymbol{r}_e | e_{x/y} \bar{\phi}_j \rangle = \frac{1}{\sqrt{2}} \langle \bar{a}_1 | \boldsymbol{r} | \bar{\phi}_j \rangle \qquad (Eq.S44)$$

Hence, the cross section is the same for all three sublevels of the ${}^{3}E$ state of VV^0.

To investigate the effect of photon polarization, we express the photon wavevector in polar coordinates (Fig. S2B) (*58*) with the *z* direction parallel to the *c* axis of 4H-SiC as:

$$\mathbf{q} = (\sin\theta\cos\varphi, \sin\theta\sin\varphi, \cos\theta) \qquad (Eq.S45)$$

and two polarization vectors in the *x*-*y* plane and the **q**-*z* plane as

$$\boldsymbol{e}_{\parallel} = (-\sin\varphi, \cos\varphi, 0) \qquad (Eq.S46)$$

$$\boldsymbol{e}_{\perp} = (-\cos\theta\cos\varphi, -\cos\theta\sin\varphi, \sin\theta) \qquad (Eq.S47)$$

As a result, any polarization vector can be expressed as a linear combination of $\boldsymbol{e}_{\parallel}$ and $\boldsymbol{e}_{\perp}$

$$\mathbf{e} = \cos\delta\, \boldsymbol{e}_{\parallel} + \sin\delta\, \boldsymbol{e}_{\perp} \qquad (Eq.S48)$$

and the square of the optical matrix element r^2 becomes

$$r^2 = |\boldsymbol{e}\cdot\boldsymbol{r}|^2 = \left| cos\,\delta\ \boldsymbol{e}_{\parallel}\cdot\boldsymbol{r} + sin\,\delta\, \boldsymbol{e}_{\perp}\cdot\boldsymbol{r}\right|^2 \qquad (Eq.S49)$$

where the angle δ is defined in Fig. S2B.

ii) Computational parameters

a. Energetics of the ionization of VV^0 to VV^-

We carried out DFT calculations with the Quantum Espresso code (*51*, *59*, *60*) and a dielectric dependent hybrid (DDH) functional (*50*), using a fraction of exact exchange equal to 0.15, corresponding to the inverse of the experimental dielectric constant of 4H-SiC in the direction parallel to the c-axis (see Fig. S2A) (*61*). We used supercells with 400 atoms (5*5*2) and sampled the supercell Brillouin zone with the Γ point. We employed SG15 ONCV pseudopotentials (*52*) and a plane-wave basis with a kinetic energy cut-off of 80 Ry. We used constrained occupations in DFT calculations to evaluate the excited states of the VV^0 defect and we computed the Freysoldt correction term (see section i) a.) with the sxdefectalign package (*53*). However, larger cell sizes are required to converge the computed values of the ZPL and here we report the values of the ZPL energy extrapolated to the values obtained with a 1024 atom-site supercell (8*8*2) (*55*).

b. Cross sections

We used the refractive index $n = 2.55$ of 4H-SiC (*62*) in our calculation of ionization and stimulated-emission cross sections and in Fig. S4 we aligned the computed (1.196 eV) and the experimental (1.096 eV) ZPL energies for the stimulated cross section and the computed (1.029 eV) and the experimental (0.88 eV) onset for the ionization cross section.

We computed spectral functions (Eq. S36) using the generating function approach and the displaced harmonic oscillator approximation (*55*). We used Gaussian functions with a standard deviation of 5 meV to broaden the δ function entering the expression of the spectral densities (Eqs. 11 and 12 of Ref. (*55*)). In the case of the stimulated emission, we broadened the ZPL with $\lambda = 0.002$ meV (Eq. 8 of Ref. (*55*)), with the temperature set to 0 K in all calculations. We used Gaussian functions with a standard deviation of 30 meV to broaden the δ function entering Eq. S39 (ionization cross section).

We computed the phonon modes of defective solids using the frozen phonon approach and a (5*5*2) supercell, with configurations generated with the PHONOPY package (*57*) and a displacement of 0.01 Å from equilibrium positions. We extrapolated our results to the (16*16*5) supercell values, as proposed in Ref. (*55*). The ground-state phonons of the *hh*-VV^0 defect (*hh*-VV^-) were used in the calculation of spectral functions of the stimulated emission (ionization). We used equal occupations of the e_x and the e_y orbitals in the phonon calculations for *hh*-VV^- to maintain the C_{3v} symmetry of the point defect.

c. Optical matrix elements

We computed optical matrix elements between Kohn-Sham orbitals for *hh*-VV^0 in its ground state $^3A_2$ as

$$\langle\psi_a|\boldsymbol{r}|\psi_b\rangle = \frac{\langle\psi_a|[\hat{H},\boldsymbol{r}]|\psi_b\rangle}{\varepsilon_a - \varepsilon_b} \qquad (Eq. S50)$$

Here $\hat{H}$ is the Kohn-Sham Hamiltonian; ε_a (ε_b) is the energy of the Kohn-Sham orbital with single particle wavefunction ψ_a (ψ_b), and the contribution of the non-local potential to the commutator is explicitly taken into account. Calculations were performed with the WEST code (*63*) at the PBE level of theory (*54*). We checked convergence as a function of the supercell size by carrying out calculations with a (5*5*2) supercell with 400 atomic sites up to a (9*9*2) supercell with 1296 atomic sites (see Fig. S6 for details).

iii) Results

a. Polarization dependence

To determine the dependence of stimulated emission and ionization spectra on the light polarization, we considered the two limiting cases of light propagation along the z-axis, parallel to the 3-fold rotational axis of the defect, which has C_{3v} symmetry, and that of propagation along the x-axis (Fig. S2). In the case of propagation along the z-axis, we did not observe any polarization dependence of either the stimulated emission or the ionization cross sections (Fig. S5A), due to the symmetry of the defect orbitals in the x-y plane orthogonal to the defect 3-fold rotational axis. In the case of light propagation within the x-y plane, we observed instead a strong polarization dependence of the cross sections (Fig. S5C). For out-of-plane polarization parallel to the defect axis, the stimulated emission vanishes, and the cross-section ratio displays a pronounced pole.

b. Convergence of calculations

To investigate the finite-size effects on computed optical matrix elements and thus on the cross sections, we performed calculations using supercells with sizes increasing from (5*5*2) to (9*9*2) (Fig. S6). We observed an increase of the stimulated emission cross section and a

decrease of the ionization cross section as a function of supercell size and reached convergence for the (9*9*2) supercell.

X. Comparisons with experiment

We note that the calculated ionization cross sections for VV^0 are almost one order of magnitude greater than for the NV^- in diamond (*40*). Our ionization wavelength in this work lies within the cross-section ratio peak corresponding to the scenario in Fig. S4B. Choosing an ionization laser closer to the ZPL would likely result in a higher ratio and a reduced effect of stimulated emission. In our experiments, no polarization dependence of the ionization laser is seen with light incident along the *c*-axis, as reproduced in Fig. S5A. Direct comparisons of the calculated and experimental ionization cross sections can be inferred from the reported rates of two-photon ionization and stimulated emission and assuming a ~1 μm spot size. To calculate the cross-section, we use the relation $R = \frac{P\sigma}{A\hbar\omega}$ where R is the measured rate, P is the optical power, A is the optical spot size area, and $\hbar\omega$ is the energy of a single photon. However, our estimated cross-sections are almost one order of magnitude lower than the calculated values. This has many possible explanations. First, the defect's quantum efficiency (QE) is non-unity, where a reduction in radiative pathways reduces the measured stimulated emission rate and competes with photoionization processes. Second, the creation of VV^- plus a hole at the VBM doesn't always result in the defect being permanently ionized. Immediate hole recapture by the VV^-, reconverting to VV^0 may happen frequently, reducing the effective "ionization" rate. The defect only becomes dark if the hole becomes trapped at a different defect or recombines elsewhere.

Some of these factors are accounted for in the ratio of cross sections, which also eliminates actual laser power and spot size from our comparisons. We report a ratio $\frac{a}{\beta} \sim 2.7$ which determines the SCC fidelity. In computation, we calculate that $\frac{\sigma_i}{\sigma_s}$ is between 2.6 and 0.9 from 1140 nm to 1150 nm. As a result, the computation is very sensitive to small errors in terms of this ratio. A direct comparison between $\frac{a}{\beta}$ and $\frac{\sigma_i}{\sigma_s}$ is difficult, although they represent similar physics. While a directly relates to the photoionization cross section, it may not relate to the actual rate of ionization as mentioned above. On the other hand, β is the modification of the spin flip rate, which is proportional to the stimulated emission cross section, but is likely also dependent on the branching ratios and intrinsic lifetime of the excited state. This makes direct comparisons to Fig. 4E difficult as mentioned above. That being said, the metric $\frac{\sigma_i}{\sigma_s}$ is directly proportional to $\frac{\alpha}{\beta}$, meaning that from a computational perspective one can still optimize the performance of SCC by optimizing and understanding $\frac{\sigma_i}{\sigma_s}$. Further studies may be able to definitively link these two factors.

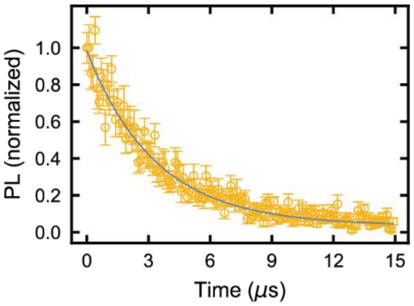

Fig. S1.
Spin flip lifetime. PL decay trace taken using time-tagging while only the E_x optical transition is excited after initializing to m_s=0. We model the decay in the PL signal as $A * \exp\left(-\frac{t}{\tau_{sf}}\right) + y_0$ (blue line fit), where A is the PL emission rate, y_0 is the laser scatter and background count rate, and τ_{sf} is the spin flip lifetime, which we calculate to be 3.3 ± 0.1 μs. The spin flip rate R_{sf} is $1/\tau_{sf}$. All data is taken at B=18 G and T=5 K. All reported errors represent 1 SD from the fit and all error bars represent 1 SD of the raw data.

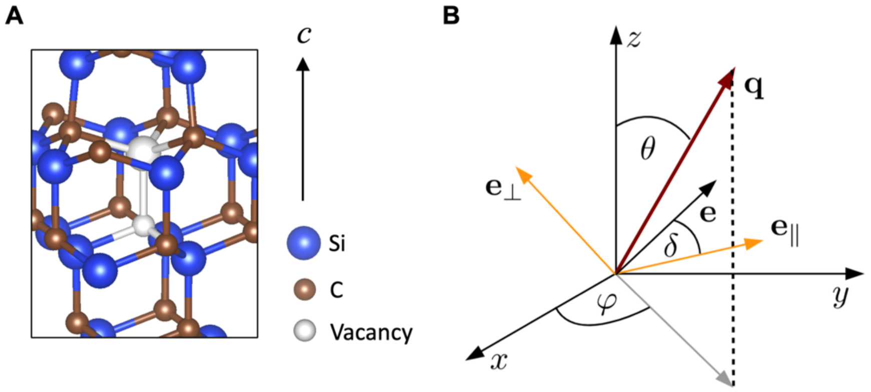

Fig. S2.
Schematic representation of the system. (**A**) Structure of the *hh* divacancy center in 4H-SiC. The 3-fold rotational axis of the divacancy center is parallel to the *c* -axis as shown in the plot. (**B**) Polarization for a photon with wavevector **q**, defined by the angles θ and φ in polar coordinates. The *z*-axis is chosen such that it aligns with the *c*-axis of 4H-SiC. $\mathbf{e}_{\parallel}$ is the component of the photon polarization vector that lies in the *x*-*y* plane; $\mathbf{e}_{\perp}$ is the out-of-plane component of the photon polarization; both $\mathbf{e}_{\parallel}$ and $\mathbf{e}_{\perp}$ are perpendicular to **q**. Any polarization direction **e** can be expressed as a linear combination of $\mathbf{e}_{\perp}$ and $\mathbf{e}_{\parallel}$.

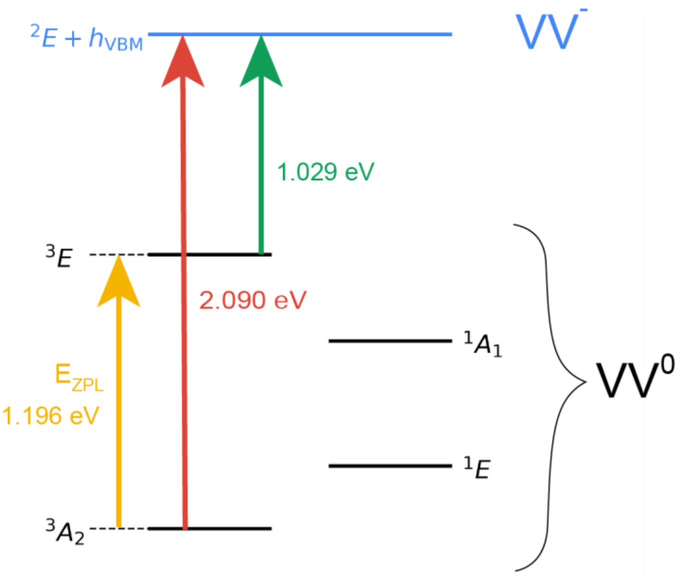

Fig. S3.

Schematic representation of the ionization of VV^0 to VV^-, where the energy of the transitions from many-body states (black lines) are indicated. $^3A_2$ and ^3E are the ground and excited triplet states of VV^0 respectively and ^2E corresponds to the ground state of VV^-; $^1A_1$ and ^1E, the singlet states of VV^0 are also indicated.

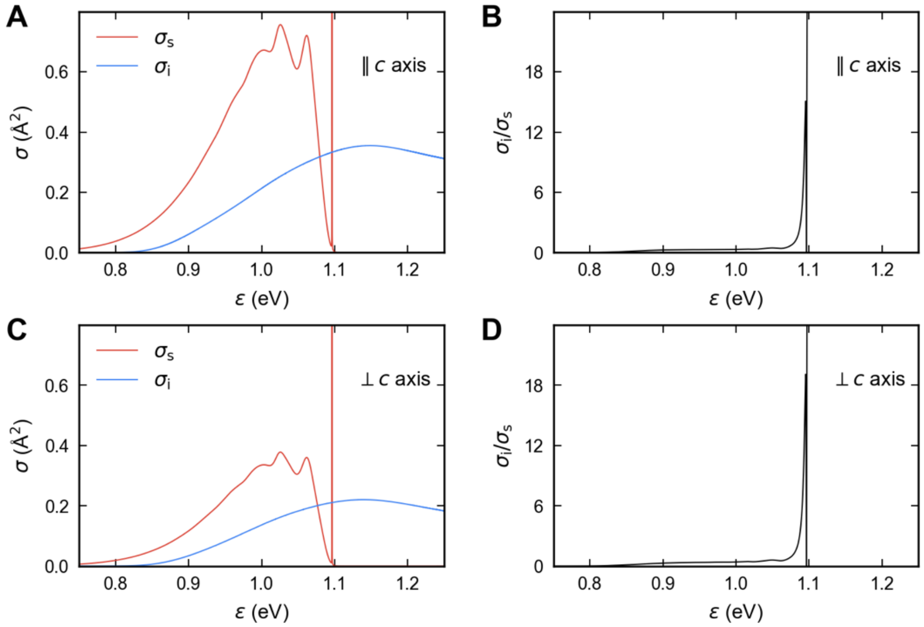

Fig. S4.
Computed stimulated emission (σ_s), ionization (σ_i) cross sections and their ratio. Computed cross sections with the light parallel (**A**) and perpendicular (**C**) to the *c* axis of 4H-SiC averaged over all possible polarization directions. Ratio of the stimulated emission and ionization cross sections with the light parallel (**B**) and perpendicular (**D**) to the *c* axis of 4H-SiC averaged over all possible polarization directions.

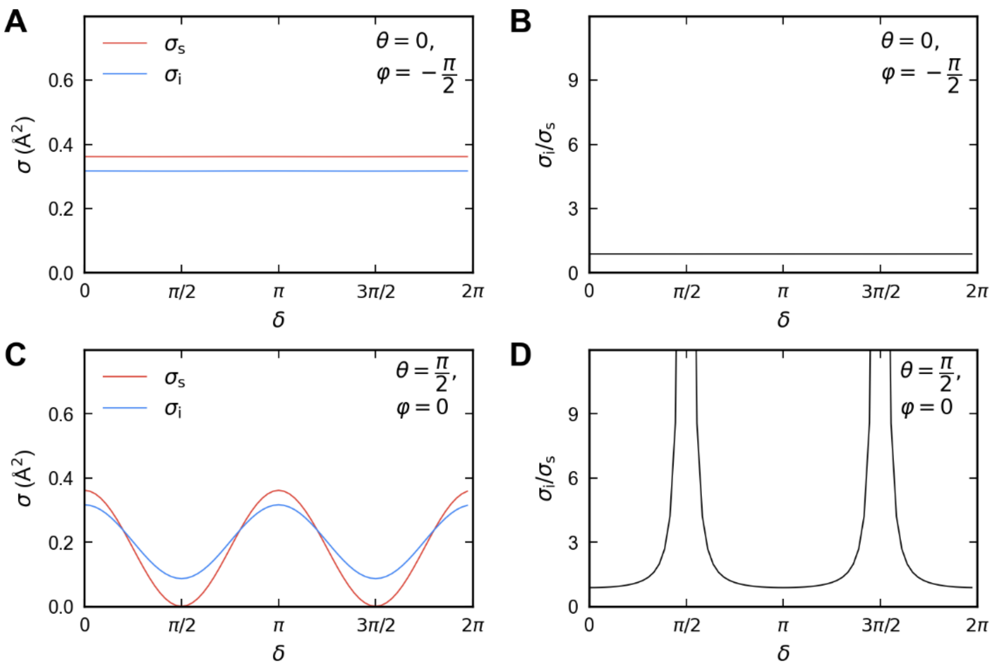

Fig. S5.
Computed stimulated emission (σ_s), ionization (σ_i) cross sections and their ratio at 1151 nm. (**A**) Computed cross sections at $\theta = 0$ and $\varphi = -\frac{\pi}{2}$ (light parallel to the *z* axis) (**C**) and at $\theta = \frac{\pi}{2}$ and $\varphi = 0$ (light perpendicular to the *x* axis) as a function of the polarization angle δ. (**B**) Ratio of the stimulated emission cross sections and ionization cross sections at $\theta = 0$ and $\varphi = -\frac{\pi}{2}$ (light parallel to the *z* axis) (**D**) and at $\theta = \frac{\pi}{2}$ and $\varphi = 0$ (light perpendicular to the *x* axis) as a function of the polarization angle δ.

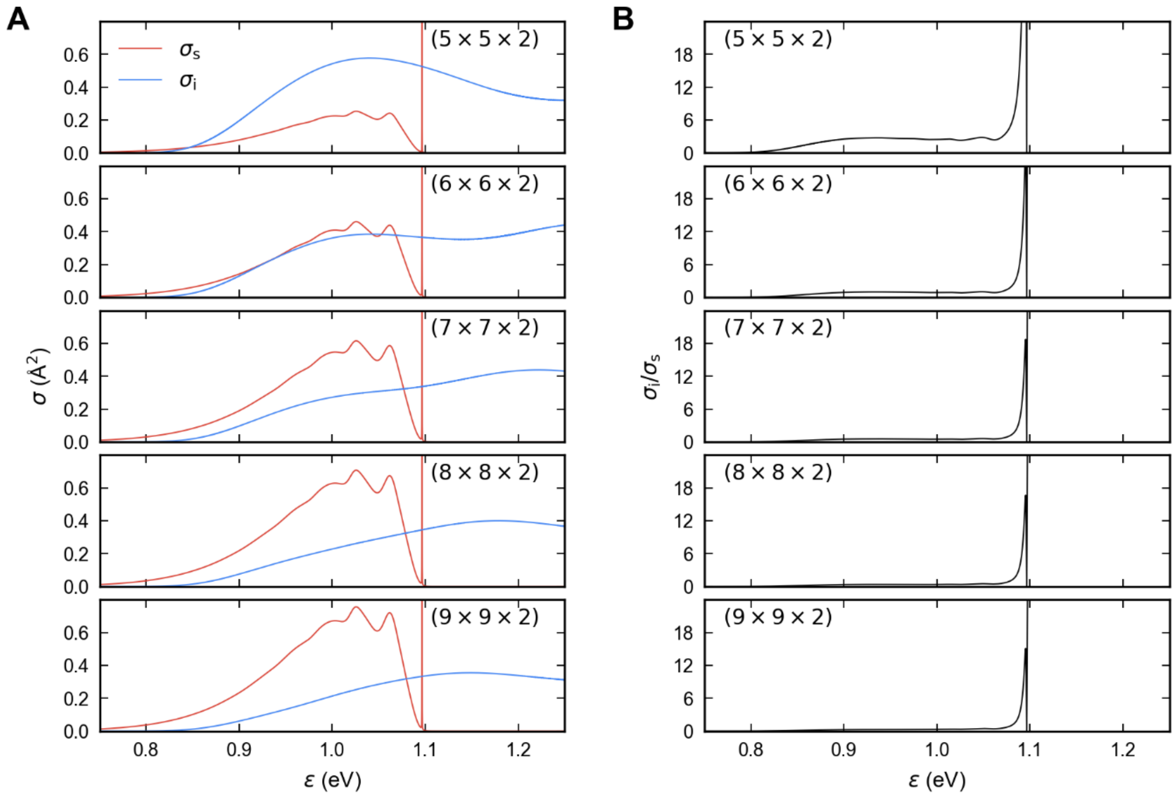

Fig. S6.
Computed stimulated emission (σ_s), ionization (σ_i) cross sections and their ratio as a function of the photon energy for supercells with different sizes. (**A**) Cross sections (**B**) and their ratio reach convergence for the (9*9*2) supercell.

Transition	Computed (DFT, eV)	Measured (eV)
$^3A_2 \rightarrow {}^3E$ (ZPL)	1.196	1.096
$^3A_2 \rightarrow {}^2E$	2.090	1.98
$^3E \rightarrow {}^2E$	1.029	0.88

Table S1.

Comparison between DFT results and experiment. Experimental values of each transition are calculated using the measured fundamental gap (3.285 eV), experimental ZPL (1.096 eV), and experimental threshold of ionization (1.31 eV) reported in Ref. (*37*).

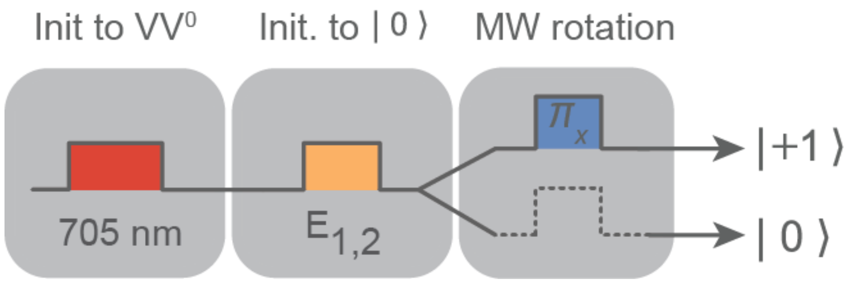

Fig. S7.
Protocol for preparation into different spin states. Both cases begin with preparation into VV^0 with the application of a 10 millisecond 705 nm charge reset pulse. Pumping of the $E_{1,2}$ lines initializes the spin to m_s=0. Further manipulation with a microwave π pulse rotates the spin to initialize into ms=+1. From there, other processes such as spin-dependent ionization via SCC, or charge readout can be performed.

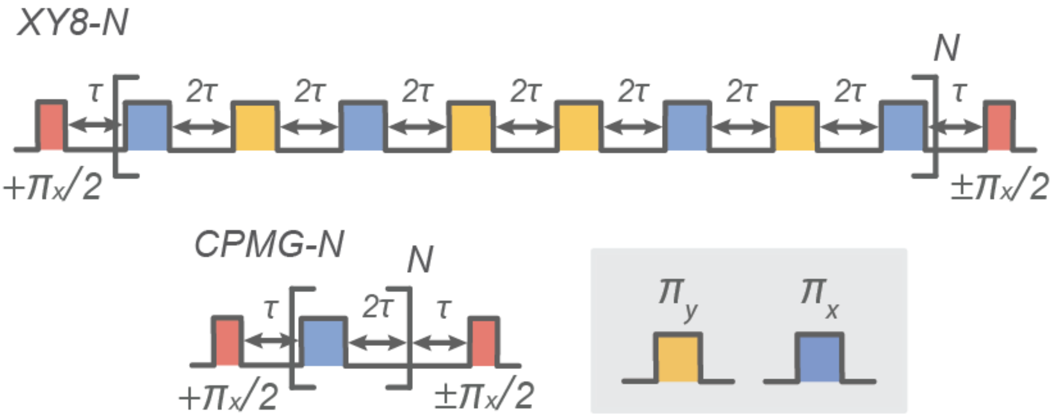

Fig. S8.
Dynamical decoupling sequences. XY8-N and CPMG-N sequences used for the coherence preservation demonstrated in the main text (Fig. 5). The final projective $\pi_x/2$ pulse employed in both sequences can have positive or negative phase to project onto m_s=+1 or m_s=0, respectively.

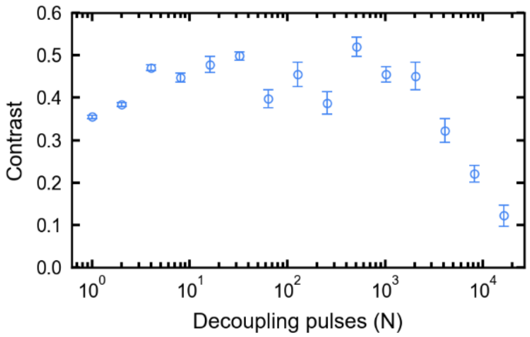

Fig. S9.
Maximum contrast with increasing pulse number. At high pulse number, the maximum contrast (at τ~0) deteriorates due to control infidelity. All error bars represent 1 SD of the raw data.

REFERENCES AND NOTES

1. H. Seo, A. L. Falk, P. V. Klimov, K. C. Miao, G. Galli, D. D. Awschalom, Quantum decoherence dynamics of divacancy spins in silicon carbide. *Nat. Commun.* **7**, 12935 (2016).

2. K. C. Miao, J. P. Blanton, C. P. Anderson, A. Bourassa, A. L. Crook, G. Wolfowicz, H. Abe, T. Ohshima, D. D. Awschalom, Universal coherence protection in a solid-state spin qubit. *Science* **369**, 1493–1497 (2020).

3. D. J. Christle, A. L. Falk, P. Andrich, P. V. Klimov, J. U. Hassan, N. T. Son, E. Janzén, T. Ohshima, D. D. Awschalom, Isolated electron spins in silicon carbide with millisecond coherence times. *Nat. Mater.* **14**, 160–163 (2015).

4. S. Kanai, F. J. Heremans, H. Seo, G. Wolfowicz, C. P. Anderson, S. E. Sullivan, G. Galli, D. D. Awschalom, H. Ohno, Generalized scaling of spin qubit coherence in over 12,000 host materials. arXiv:2102.02986 [quant-ph] (2021); http://arxiv.org/abs/2102.02986.

5. D. J. Christle, P. V. Klimov, C. F. de las Casas, K. Szász, V. Ivády, V. Jokubavicius, J. U. Hassan, M. Syväjärvi, W. F. Koehl, T. Ohshima, N. T. Son, E. Janzén, Á. Gali, D. D. Awschalom, Isolated spin qubits in SiC with a high-fidelity infrared spin-to-photon interface. *Phys. Rev. X* **7**, 021046 (2017).

6. K. C. Miao, A. Bourassa, C. P. Anderson, S. J. Whiteley, A. L. Crook, S. L. Bayliss, G. Wolfowicz, G. Thiering, P. Udvarhelyi, V. Ivády, H. Abe, T. Ohshima, Á. Gali, D. D. Awschalom, Electrically driven optical interferometry with spins in silicon carbide. *Sci. Adv.* **5**, eaay0527 (2019).

7. A. Bourassa, C. P. Anderson, K. C. Miao, M. Onizhuk, H. Ma, A. L. Crook, H. Abe, J. Ul-Hassan, T. Ohshima, N. T. Son, G. Galli, D. D. Awschalom, Entanglement and control of single nuclear spins in isotopically engineered silicon carbide. *Nat. Mater.* **19**, 1319–1325 (2020).

8. G. Wolfowicz, F. J. Heremans, C. P. Anderson, S. Kanai, H. Seo, A. Gali, G. Galli, D. D. Awschalom, Quantum guidelines for solid-state spin defects. *Nat. Rev. Mater.* **6**, 906–925 (2021).

9. A. L. Crook, C. P. Anderson, K. C. Miao, A. Bourassa, H. Lee, S. L. Bayliss, D. O. Bracher, X. Zhang, H. Abe, T. Ohshima, E. L. Hu, D. D. Awschalom, Purcell enhancement of a single silicon carbide color center with coherent spin control. *Nano Lett.* **20**, 3427–3434 (2020).

10. D. M. Lukin, C. Dory, M. A. Guidry, K. Y. Yang, S. D. Mishra, R. Trivedi, M. Radulaski, S. Sun, D. Vercruysse, G. H. Ahn, J. Vučković, 4H-silicon-carbide-on-insulator for integrated quantum and nonlinear photonics. *Nat. Photonics* **14**, 330–334 (2020).

11. C. P. Anderson, A. Bourassa, K. C. Miao, G. Wolfowicz, P. J. Mintun, A. L. Crook, H. Abe, J. U. Hassan, N. T. Son, T. Ohshima, D. D. Awschalom, Electrical and optical control of single spins integrated in scalable semiconductor devices. *Science* **366**, 1225–1230 (2019).

12. S. J. Whiteley, G. Wolfowicz, C. P. Anderson, A. Bourassa, H. Ma, M. Ye, G. Koolstra, K. J. Satzinger, M. V. Holt, F. J. Heremans, A. N. Cleland, D. I. Schuster, G. Galli, D. D. Awschalom, Spin–phonon interactions in silicon carbide addressed by Gaussian acoustics. *Nat. Phys.* **15**, 490–495 (2019).

13. S. J. Whiteley, F. J. Heremans, G. Wolfowicz, D. D. Awschalom, M. V. Holt, Correlating dynamic strain and photoluminescence of solid-state defects with stroboscopic x-ray diffraction microscopy. *Nat. Commun.* **10**, 3386 (2019).

14. L. Robledo, L. Childress, H. Bernien, B. Hensen, P. F. A. Alkemade, R. Hanson, High-fidelity projective read-out of a solid-state spin quantum register. *Nature* **477**, 574–578 (2011).

15. M. Raha, S. Chen, C. M. Phenicie, S. Ourari, A. M. Dibos, J. D. Thompson, Optical quantum nondemolition measurement of a single rare earth ion qubit. *Nat. Commun.* **11**, 1605 (2020).

16. N. T. Son, C. P. Anderson, A. Bourassa, K. C. Miao, C. Babin, M. Widmann, M. Niethammer, J. Ul Hassan, N. Morioka, I. G. Ivanov, F. Kaiser, J. Wrachtrup, D. D. Awschalom, Developing silicon carbide for quantum spintronics. *Appl. Phys. Lett.* **116**, 190501 (2020).

17. H. Bernien, B. Hensen, W. Pfaff, G. Koolstra, M. S. Blok, L. Robledo, T. H. Taminiau, M. Markham, D. J. Twitchen, L. Childress, R. Hanson, Heralded entanglement between solid-state qubits separated by three metres. *Nature* **497**, 86–90 (2013).

18. J. Cramer, N. Kalb, M. A. Rol, B. Hensen, M. S. Blok, M. Markham, D. J. Twitchen, R. Hanson, T. H. Taminiau, Repeated quantum error correction on a continuously encoded qubit by real-time feedback. *Nat. Commun.* **7**, 11526 (2016).

19. D. M. Irber, F. Poggiali, F. Kong, M. Kieschnick, T. Lühmann, D. Kwiatkowski, J. Meijer, J. Du, F. Shi, F. Reinhard, Robust all-optical single-shot readout of nitrogen-vacancy centers in diamond. *Nat. Commun.* **12**, 532 (2021).

20. Q. Zhang, Y. Guo, W. Ji, M. Wang, J. Yin, F. Kong, Y. Lin, C. Yin, F. Shi, Y. Wang, J. Du, High-fidelity single-shot readout of single electron spin in diamond with spin-to-charge conversion. *Nat. Commun.* **12**, 1529 (2021).

21. J. R. Maze, P. L. Stanwix, J. S. Hodges, S. Hong, J. M. Taylor, P. Cappellaro, L. Jiang, M. V. G. Dutt, E. Togan, A. S. Zibrov, A. Yacoby, R. L. Walsworth, M. D. Lukin, Nanoscale magnetic sensing with an individual electronic spin in diamond. *Nature* **455**, 644–647 (2008).

22. J. M. Taylor, P. Cappellaro, L. Childress, L. Jiang, D. Budker, P. R. Hemmer, A. Yacoby, R. Walsworth, M. D. Lukin, High-sensitivity diamond magnetometer with nanoscale resolution. *Nat. Phys.* **4**, 810–816 (2008).

23. C. L. Degen, F. Reinhard, P. Cappellaro, Quantum sensing. *Rev. Mod. Phys.* **89**, 035002 (2017).

24. T. Gullion, D. B. Baker, M. S. Conradi, New, compensated Carr-Purcell sequences. *J. Magn. Reson.* **89**, 479–484 (1990).

25. H. Y. Carr, E. M. Purcell, Effects of diffusion on free precession in nuclear magnetic resonance experiments. *Phys. Rev.* **94**, 630–638 (1954).

26. J. T. Muhonen, J. P. Dehollain, A. Laucht, F. E. Hudson, R. Kalra, T. Sekiguchi, K. M. Itoh, D. N. Jamieson, J. C. McCallum, A. S. Dzurak, A. Morello, Storing quantum information for 30 seconds in a nanoelectronic device. *Nat. Nanotechnol.* **9**, 986–991 (2014).

27. A. M. Tyryshkin, S. Tojo, J. J. L. Morton, H. Riemann, N. V. Abrosimov, P. Becker, H.-J. Pohl, T. Schenkel, M. L. W. Thewalt, K. M. Itoh, S. A. Lyon, Electron spin coherence exceeding seconds in high-purity silicon. *Nat. Mater.* **11**, 143–147 (2012).

28. M. H. Abobeih, J. Cramer, M. A. Bakker, N. Kalb, M. Markham, D. J. Twitchen, T. H. Taminiau, One-second coherence for a single electron spin coupled to a multi-qubit nuclear-spin environment. *Nat. Commun.* **9**, 2552 (2018).

29. N. Bar-Gill, L. M. Pham, A. Jarmola, D. Budker, R. L. Walsworth, Solid-state electronic spin coherence time approaching one second. *Nat. Commun.* **4**, 1743 (2013).

30. D. Simin, H. Kraus, A. Sperlich, T. Ohshima, G. V. Astakhov, V. Dyakonov, Locking of electron spin coherence above 20 ms in natural silicon carbide. *Phys. Rev. B* **95**, 161201 (2017).

31. N. T. Son, P. Carlsson, J. Ul Hassan, E. Janzén, T. Umeda, J. Isoya, A. Gali, M. Bockstedte, N. Morishita, T. Ohshima, H. Itoh, Divacancy in 4H-SiC. *Phys. Rev. Lett.* **96**, 055501 (2006).

32. P. Tamarat, N. B. Manson, J. P. Harrison, R. L. McMurtrie, A. Nizovtsev, C. Santori, R. G. Beausoleil, P. Neumann, T. Gaebel, F. Jelezko, P. Hemmer, J. Wrachtrup, Spin-flip and spin-conserving optical transitions of the nitrogen-vacancy centre in diamond. *New J. Phys.* **10**, 045004 (2008).

33. N. B. Manson, J. P. Harrison, M. J. Sellars, Nitrogen-vacancy center in diamond: Model of the electronic structure and associated dynamics. *Phys. Rev. B* **74**, 104303 (2006).

34. L. Gordon, A. Janotti, C. G. van de Walle, Defects as qubits in *3C*– and *4H*–SiC. *Phys. Rev. B* **92**, 045208 (2015).

35. G. Wolfowicz, C. P. Anderson, A. L. Yeats, S. J. Whiteley, J. Niklas, O. G. Poluektov, F. J. Heremans, D. D. Awschalom, Optical charge state control of spin defects in 4H-SiC. *Nat. Commun.* **8**, 1876 (2017).

36. D. A. Golter, C. W. Lai, Optical switching of defect charge states in *4H*-SiC. *Sci. Rep.* **7**, 13406 (2017).

37. B. Magnusson, N. T. Son, A. Csóré, A. Gällström, T. Ohshima, Á. Gali, I. G. Ivanov, Excitation properties of the divacancy in *4H*-SiC. *Phys. Rev. B.* **98**, 195202 (2018).

38. M. Bockstedte, F. Schütz, T. Garratt, V. Ivády, A. Gali, Ab initio description of highly correlated states in defects for realizing quantum bits. *npj Quant. Mater.* **3**, 31 (2018).

39. D. A. Hopper, H. J. Shulevitz, L. C. Bassett, Spin readout techniques of the nitrogen-vacancy center in diamond. *Micromachines* **9**, 10.3390/mi9090437 (2018).

40. L. Razinkovas, M. Maciaszek, F. Reinhard, M. W. Doherty, A. Alkauskas, Photoionization of negatively charged NV centers in diamond: Theory and ab initio calculations. *Phys. Rev. B* **104**, 235301 (2021).

41. M. J. Biercuk, H. Bluhm, Phenomenological study of decoherence in solid-state spin qubits due to nuclear spin diffusion. *Phys. Rev. B* **83**, 235316 (2011).

42. J. Medford, Ł. Cywiński, C. Barthel, C. M. Marcus, M. P. Hanson, A. C. Gossard, Scaling of dynamical decoupling for spin qubits. *Phys. Rev. Lett.* **108**, 086802 (2012).

43. G. Wolfowicz, C. P. Anderson, B. Diler, O. G. Poluektov, F. J. Heremans, D. D. Awschalom, Vanadium spin qubits as telecom quantum emitters in silicon carbide. *Sci. Adv.* **6**, eaaz1192 (2020).

44. B. Diler, S. J. Whiteley, C. P. Anderson, G. Wolfowicz, M. E. Wesson, E. S. Bielejec, F. Joseph Heremans, D. D. Awschalom, Coherent control and high-fidelity readout of chromium ions in commercial silicon carbide. *npj Quantum Inf.* **6**, 11 (2020).

45. R. Nagy, M. Niethammer, M. Widmann, Y.-C. Chen, P. Udvarhelyi, C. Bonato, J. U. Hassan, R. Karhu, I. G. Ivanov, N. T. Son, J. R. Maze, T. Ohshima, Ö. O. Soykal, Á. Gali, S.-Y. Lee, F. Kaiser, J. Wrachtrup, High-fidelity spin and optical control of single silicon-vacancy centres in silicon carbide. *Nat. Commun.* **10**, 1954 (2019).

46. J. Jeske, D. W. M. Lau, X. Vidal, L. P. McGuinness, P. Reineck, B. C. Johnson, M. W. Doherty, J. C. McCallum, S. Onoda, F. Jelezko, T. Ohshima, T. Volz, J. H. Cole, B. C. Gibson, A. D. Greentree, Stimulated emission from nitrogen-vacancy centres in diamond. *Nat. Commun.* **8**, 14000 (2017).

47. G. Wolfowicz, C. P. Anderson, S. J. Whiteley, D. D. Awschalom, Heterodyne detection of radio-frequency electric fields using point defects in silicon carbide. *Appl. Phys. Lett.* **115**, 043105 (2019).

48. D. R. Candido, M. E. Flatté, Suppression of the optical linewidth and spin decoherence of a quantum spin center in a *p-i-n* diode. arXiv:2008.13289 [cond-mat.mes-hall] (2020); https://arxiv.org/abs/2008.13289.

49. K. Ghosh, H. Ma, M. Onizhuk, V. Gavini, G. Galli, Spin–spin interactions in defects in solids from mixed all-electron and pseudopotential first-principles calculations. *npj Comput. Mater.* **7**, 123 (2021).

50. J. H. Skone, M. Govoni, G. Galli, Self-consistent hybrid functional for condensed systems. *Phys. Rev. B* **89**, 195112 (2014).

51. P. Giannozzi, S. Baroni, N. Bonini, M. Calandra, R. Car, C. Cavazzoni, D. Ceresoli, G. L. Chiarotti, M. Cococcioni, I. Dabo, A. Dal Corso, S. de Gironcoli, S. Fabris, G. Fratesi, R. Gebauer, U. Gerstmann, C. Gougoussis, A. Kokalj, M. Lazzeri, L. Martin-Samos, N. Marzari, F. Mauri, R. Mazzarello, S. Paolini, A. Pasquarello, L. Paulatto, C. Sbraccia, S. Scandolo, G. Sclauzero, A. P. Seitsonen, A. Smogunov, P. Umari, R. M. Wentzcovitch, QUANTUM ESPRESSO: A modular and open-source software project for quantum simulations of materials. *J. Phys. Condens. Matter* **21**, 395502 (2009).

52. M. Schlipf, F. Gygi, Optimization algorithm for the generation of ONCV pseudopotentials. *Comput. Phys. Commun.* **196**, 36–44 (2015).

53. C. Freysoldt, J. Neugebauer, C. G. van de Walle, Fully *Ab Initio* finite-size corrections for charged-defect supercell calculations. *Phys. Rev. Lett.* **102**, 016402 (2009).

54. J. P. Perdew, K. Burke, M. Ernzerhof, Generalized gradient approximation made simple. *Phys. Rev. Lett.* **77**, 3865–3868 (1996).

55. Y. Jin, M. Govoni, G. Wolfowicz, S. E. Sullivan, F. J. Heremans, D. D. Awschalom, G. Galli, Photoluminescence spectra of point defects in semiconductors: Validation of first-principles calculations. *Phys. Rev. Materials.* **5**, 84603 (2021).

56. L. Razinkovas, M. W. Doherty, N. B. Manson, C. G. de Walle, A. Alkauskas, Vibrational and vibronic structure of isolated point defects: The nitrogen-vacancy center in diamond. *Phys. Rev. B.* **104**, 45303 (2021).

57. A. Togo, I. Tanaka, First principles phonon calculations in materials science. *Scr. Mater.* **108**, 1–5 (2015).

58. H.-Y. Chen, M. Palummo, D. Sangalli, M. Bernardi, Theory and ab initio computation of the anisotropic light emission in monolayer transition metal dichalcogenides. *Nano Lett.* **18**, 3839–3843 (2018).

59. P. Giannozzi, O. Andreussi, T. Brumme, O. Bunau, M. B. Nardelli, M. Calandra, R. Car, C. Cavazzoni, D. Ceresoli, M. Cococcioni, N. Colonna, I. Carnimeo, A. D. Corso, S. de Gironcoli, P. Delugas, R. A. DiStasio, A. Ferretti, A. Floris, G. Fratesi, G. Fugallo, R. Gebauer, U. Gerstmann, F. Giustino, T. Gorni, J. Jia, M. Kawamura, H.-Y. Ko, A. Kokalj, E. Küçükbenli, M. Lazzeri, M. Marsili, N. Marzari, F. Mauri, N. L. Nguyen, H.-V. Nguyen, A. Otero-de-la-Roza, L. Paulatto, S. Poncé, D. Rocca, R. Sabatini, B. Santra, M. Schlipf, A. P. Seitsonen, A. Smogunov, I. Timrov, T. Thonhauser, P. Umari, N. Vast, X. Wu, S. Baroni, Advanced capabilities for materials modelling with Quantum ESPRESSO. *J. Phys. Condens. Matter* **29**, 465901 (2017).

60. P. Giannozzi, O. Baseggio, P. Bonfà, D. Brunato, R. Car, I. Carnimeo, C. Cavazzoni, S. de Gironcoli, P. Delugas, F. F. Ruffino, A. Ferretti, N. Marzari, I. Timrov, A. Urru, S. Baroni, Quantum ESPRESSO toward the exascale. *J. Chem. Phys.* **152**, 154105 (2020).

61. L. Patrick, W. J. Choyke, Static dielectric constant of SiC. *Phys. Rev. B.* **2**, 15 (1970).

62. Y. Goldberg, M. Levinshtein, S. Rumyantsev, *Properties of Advanced Semiconductor Materials: GaN, AIN, InN, BN, SiC, SiGe* (John Wiley & Sons, 2001).

63. M. Govoni, G. Galli, Large scale GW calculations. *J. Chem. Theory Comput.* **11**, 2680–2696 (2015).
